# The Globular C1q Receptor Is Required for Epidermal Growth Factor Receptor Signaling during Candida albicans Infection

**DOI:** 10.1128/mBio.02716-21

**Published:** 2021-11-02

**Authors:** Quynh T. Phan, Jianfeng Lin, Norma V. Solis, Michael Eng, Marc Swidergall, Feng Wang, Shan Li, Sarah L. Gaffen, Tsui-Fen Chou, Scott G. Filler

**Affiliations:** a Institute for Infection and Immunity, Lundquist Institute for Biomedical Innovation at Harbor-UCLA Medical Center, Torrance, California, USA; b David Geffen School of Medicine at UCLA, Los Angeles, California, USA; c Department of Pediatrics, Lundquist Institute for Biomedical Innovation at Harbor-UCLA Medical Center, Torrance, California, USA; d Division of Rheumatology and Clinical Immunology, University of Pittsburgh, Pittsburgh, Pennsylvania, USA; Duke University Medical Center

**Keywords:** *Candida albicans*, oral epithelial cells, endocytosis, epidermal growth factor receptor, host defense

## Abstract

During oropharyngeal candidiasis, Candida albicans activates the epidermal growth factor receptor (EGFR), which induces oral epithelial cells to endocytose the fungus and synthesize proinflammatory mediators. To elucidate EGFR signaling pathways that are stimulated by C. albicans, we used proteomics to identify 1,214 proteins that were associated with EGFR in C. albicans-infected cells. Seven of these proteins were selected for additional study. Among these proteins, WW domain-binding protein 2, Toll-interacting protein, interferon-induced transmembrane protein 3 (IFITM3), and the globular C1q receptor (gC1qR) were found to associate with EGFR in viable oral epithelial cells. Each of these proteins was required for maximal endocytosis of C. albicans, and all regulated fungus-induced production of interleukin-1β (IL-1β) and/or IL-8, either positively or negatively. gC1qR was found to function as a key coreceptor with EGFR. Interacting with the C. albicans Als3 invasin, gC1qR was required for the fungus to induce autophosphorylation of both EGFR and the ephrin type A receptor 2. The combination of gC1qR and EGFR was necessary for maximal endocytosis of C. albicans and secretion of IL-1β, IL-8, and granulocyte-macrophage colony-stimulating factor (GM-CSF) by human oral epithelial cells. In mouse oral epithelial cells, inhibition of gC1qR failed to block C. albicans-induced phosphorylation, and knockdown of IFITM3 did not inhibit C. albicans endocytosis, indicating that gC1qR and IFITM3 function differently in mouse versus human oral epithelial cells. Thus, this work provides an atlas of proteins that associate with EGFR and identifies several that play a central role in the response of human oral epithelial cells to C. albicans infection.

## INTRODUCTION

Candida albicans grows as a harmless commensal in the oral cavity of at least 50% of normal adults (1). However, when either local or systemic immune defenses are weakened, C. albicans can proliferate and cause oropharyngeal candidiasis (2). This disease causes substantial morbidity in patients with HIV/AIDS, dentures, organ transplantation, cancer, diabetes, and xerostomia (3–6). Although most patients with oropharyngeal candidiasis readily respond to treatment with antifungal agents such as fluconazole during their first episode of infection, patients with recurrent disease are at risk for developing infection with an azole-resistant strain (7, 8).

During oropharyngeal candidiasis, C. albicans invades the epithelial cell lining of the oropharynx, stimulating a strong proinflammatory host response that is driven by interleukin-17 (IL-17) and IL-22 signaling, which is initiated from innate lymphocytes and subsequently from the adaptive immune response (9, 10). When C. albicans adheres to an epithelial cell, it interacts with and activates multiple epithelial cell receptors, including the ephrin type A receptor 2 (EphA2), E-cadherin, the platelet-derived growth factor receptor BB, HER2, and the epidermal growth factor receptor (EGFR) (11–13). Among these receptors, EGFR plays a central role in triggering the epithelial cell endocytosis of C. albicans and stimulating a proinflammatory response. EGFR interacts with EphA2, HER2, E-cadherin, Src family kinases, and the aryl hydrocarbon receptor, triggering changes in the actin cytoskeleton that lead to the endocytosis of the fungus (11, 13, 14). EGFR also interacts with EphA2 to stimulate epithelial cells to secrete proinflammatory mediators, including defensins, IL-1α, IL-1β, CXCL8/IL-8, and CCL20 (11, 15, 16). The defensins have direct candidacidal activity, while the cytokines and chemokines recruit phagocytes to the focus of infection and enhance their capacity to kill the invading fungus.

Because of the key role of EGFR in mediating the oral epithelial cell response to C. albicans, we sought to obtain a more comprehensive view of the spectrum of host cell proteins that interact with this receptor. Using a proteomics approach, we found that EGFR associates with 1,214 epithelial cell proteins in C. albicans*-*infected cells. Of these proteins, 13 had increased association with EGFR in the C. albicans*-*infected cells relative to uninfected cells. In intact epithelial cells, four proteins associated with EGFR in the vicinity of C. albicans hyphae: WW domain-binding protein 2 (WBP2), which governs EGFR expression and signaling in cancer cells; Toll-interacting protein (TOLLlIP), which is a negative regulator of Toll-like receptor signaling; interferon-induced transmembrane protein 3 (IFITM3), which is an antiviral protein; and the globular C1q receptor (gC1qR), which is a multifunctional protein that interacts with a variety of serum components. We interrogated these proteins in the context of C. albicans infection of human oral epithelial cells, and found that each of these proteins is required for maximal endocytosis of C. albicans. Moreover, they all regulate the production of innate cytokines, such as IL-1β and/or IL-8, either positively or negatively. Additionally, gC1qR functions as a key coreceptor that is required for C. albicans to stimulate EGFR and to induce endocytosis and an epithelial cell proinflammatory response.

## RESULTS

### EGFR associates with multiple proteins that mediate endocytosis and govern actin dynamics.

To identify epithelial cell proteins that associate with EGFR, we infected the OKF6/TERT-2 oral epithelial cell line (17) with C. albicans yeast for 90 min, cross-linked the proteins with formaldehyde, and then performed immunoprecipitation of whole-cell lysates with an anti-EGFR antibody. The proteins that were associated with EGFR were then identified by liquid chromatography-tandem mass spectrometry (LC-MS/MS). As controls, we processed in parallel uninfected epithelial cells as well as epithelial cells incubated with epidermal growth factor (EGF) for 5 min. In total, 1,214 proteins were associated with EGFR in all three samples of epithelial cells infected with C. albicans (see Table S1 in the supplemental material), and 1,278 were associated with EGFR in both samples of epithelial cells that had been incubated with EGF (see Table S2 in the supplemental material). The majority of these proteins were constitutively associated with EGFR. However, 13 proteins showed at least a 2-fold increase in association with EGFR in all three samples of C. albicans*-*infected cells relative to uninfected cells (Table 1), and 37 had increased association with EGFR in the EGF-exposed cells (see Table S3 in the supplemental material). Among the proteins that had increased association with EGFR in response to C. albicans infection, four associated with EGFR only in cells exposed to C. albicans, but not EGF. Among these were WW domain-binding protein 2 (WBP2), guanylate binding protein family member 6 (GPB6), subunit J of eukaryotic translation initiation factor 3, and calcineurin subunit B type 1 (Table 1). Only three proteins were found to associate with EGFR in cells exposed to EGF but not C. albicans. One was EGF itself, and the others were galectin-9B and ERBB receptor feedback inhibitor 1 (Table S3). Collectively, these results suggest that while the profiles of proteins that associate with EGFR in response to C. albicans and EGF are similar, some proteins are uniquely recruited in response to C. albicans.

**TABLE 1:** List of proteins that had increased association with EGFR in response to infection with C. albicans

Accession no.	Gene	Protein	Result for^*a*^:				
			C. albicans replicate			EGF-incubated replicate	
			1	2	3	1	2
K7EIJ0	*WBP2*	WW domain-binding protein 2	Inf	Inf	Inf		
B4DRS8	*GBP6*	cDNA FLJ54753, highly similar to guanylate binding protein family, member 6	Inf	Inf	Inf		
B4DUI3	*EIF3J*	Eukaryotic translation initiation factor 3 subunit J	2.63	Inf	Inf		
F6U1T9	*PPP3R1*	Calcineurin subunit B type 1	Inf	2.15	Inf	0.00	
Q9H0E2	*TOLLIP*	Toll-interacting protein	Inf	Inf	Inf	Inf	
P35354	*PTGS2*	Prostaglandin G/H synthase 2	3.29	Inf	Inf		Inf
Q15075	*EEA1*	Early endosome antigen 1	15.80	12.72	3.11	6.38	1.83
P32926	*DSG3*	Desmoglein-3	3.30	5.99	2.08	0.61	0.76
Q01628	*IFITM3*	Interferon-induced transmembrane protein 3	3.17	3.18	2.68	0.00	0.91
Q14165	*MLEC*	Malectin	2.10	2.46	2.57	1.45	1.61
P02787	*TF*	Serotransferrin	4.06	2.05	2.24	2.30	1.32
P21281	*ATP6V1B2*	V-type proton ATPase subunit B, brain isoform	4.10	7.36	Inf	4.03	Inf
P36543	*ATP6V1E1*	V-type proton ATPase subunit E 1	2.47	Inf	2.26	Inf	1.94

a Shown are individual results from 3 biological replicates for epithelial cells infected with C. albicans for 90 min and 2 biological replicates from epithelial cells incubated with epidermal growth factor (EGF) for 5 min. Values are the ratio of the amount of protein detected in cells exposed to C. albicans or EGF relative to unstimulated control cells. “Inf” (i.e., infinite change”) indicates proteins that were undetectable in unstimulated control cells.

10.1128/mBio.02716-21.6 TABLE S1 List of proteins that were found to associate with the epidermal growth factor receptor (EGFR) in all three biological replicates of oral epithelial cells exposed to C. albicans for 90 min. Two samples of epithelial cells were incubated with epidermal growth factor (EGF) for 5 min. Data are presented as the fold change relative to control epithelial cells that were incubated in tissue culture medium alone. Inf, infinite change because the protein was undetectable in the control cells. Download Table S1, XLSX file, 0.1 MB. Copyright © 2021 Phan et al. 2021 Phan et al. https://creativecommons.org/licenses/by/4.0/ This content is distributed under the terms of the Creative Commons Attribution 4.0 International license.

10.1128/mBio.02716-21.7 TABLE S2 List of proteins that were found to associate with EGFR in two biological replicates of oral epithelial cells that were incubated with epidermal growth factor (EGF) for 5 min. Also shown are the proteins that associated with EGFR in 1 to 3 samples of epithelial cells infected with C. albicans. Data are presented as the fold change relative to control epithelial cells that were incubated in tissue culture medium alone. Inf, infinite change because the protein was undetectable in the control cells. Download Table S2, XLSX file, 0.1 MB. Copyright © 2021 Phan et al. 2021 Phan et al. https://creativecommons.org/licenses/by/4.0/ This content is distributed under the terms of the Creative Commons Attribution 4.0 International license.

10.1128/mBio.02716-21.8 TABLE S3 List of proteins in oral epithelial cells incubated with EGF for 5 min that were found to have increased association with EGFR in both biological replicates. Data are presented as the fold change relative to control epithelial cells that were incubated in tissue culture medium alone. Inf, infinite change because the protein was undetectable in the control cells. Download Table S3, XLSX file, 0.01 MB. Copyright © 2021 Phan et al. 2021 Phan et al. https://creativecommons.org/licenses/by/4.0/ This content is distributed under the terms of the Creative Commons Attribution 4.0 International license.

Analysis of proteins constitutively associated with EGFR provided insight into the mechanisms by which EGFR induces epithelial cells to endocytose C. albicans. We found that EGFR associated with EphA2, E-cadherin, and HER2 (Table 2), consistent with our previous findings that EGFR functions in the same pathways as these three receptors (11, 13, 18). EGFR was found to associate with two members of the Src family of kinases, Lyn and Yes, suggesting that these kinases may activate EGFR (Table 2) (14). It was notable that EGFR was associated with the lipid raft proteins caveolin-1, caveolin-2, flotillin-1, flotillin-2, arf6, and RhoA (Table 2) (19, 20), indicating that EGFR-containing complexes are likely localized to these microdomains.

**TABLE 2:** List of selected proteins that had constitutive association with EGFR in response to infection with C. albicans

Function/location	Accession no.	Gene	Protein	Result for^*a*^:				
				C. albicans replicate			EGF-incubated replicate	
				1	2	3	1	2
Receptor	P29317	*EPHA2*	Ephrin type-A receptor 2	1.65	0.68	0.44	0.58	0.33
	D3XNU5	*CDH1*	E-cadherin 1	0.48	0.79	0.64	0.43	0.42
	P04626-4	*ERBB2*	Isoform 4 of ErbB-2 (HER2)	0.63	1.57	0.93	1.04	1.02

Src tyrosine kinase	P07948-2	*LYN*	Isoform 2 of tyrosine-protein kinase Lyn	6.58	0.68	0.63	0.57	0.58
	B2RA70	*YES1*	Highly similar to Homo sapiens v-yes-1 Yamaguchi sarcoma viral oncogene homolog 1	4.23	0.65	0.53	0.75	0.46

Lipid rafts	Q03135	*CAV1*	Caveolin-1	1.62	1.22	0.87	1.19	0.83
	P51636	*CAV2*	Caveolin-2	Inf	Inf	0.76	Inf	0.46
	O75955	*FLOT1*	Flotillin-1	1.64	1.01	0.97	0.90	0.80
	Q6FG43	*FLOT2*	Flotillin-2	1.34	1.04	0.90	0.93	0.68
	P62330	*ARF6*	ADP-ribosylation factor 6	1.18	0.68	0.72	0.78	0.53
	P61586	*RHOA*	RhoA	1.90	0.96	0.66	0.89	0.77

Clathrin internalization pathway	Q00610-2	*CLTC*	Isoform 2 of clathrin heavy chain 1	1.69	0.88	0.80	2.22	1.12
	P09496-2	*CLTA*	Isoform non-brain of clathrin light chain A	4.64	1.05	1.61	2.72	1.55
	P09497-2	*CLTB*	Isoform non-brain of clathrin light chain B	Inf	0.94	1.20	2.03	1.44
	Q14247	*CTTN*	Cortactin	3.56	1.31	0.90	0.39	0.39
	B5BU72	*PICALM*	Phosphatidylinositol-binding clathrin assembly protein isoform 2	1.61	0.75	1.12	2.18	1.44

Actin-related protein 2/3 complex	P61160	*ACTR2*	Actin-related protein 2	2.96	1.12	0.94	0.61	0.50
	P61158	*ACTR3*	Actin-related protein 3	1.93	1.17	0.55	0.43	0.49
	O15143	*ARPC1B*	Actin-related protein 2/3 complex subunit 1B	2.14	1.75	1.06	0.58	0.36
	O15144	*ARPC2*	Actin-related protein 2/3 complex subunit 2	4.10	1.09	1.31	0.75	0.74
	B2R4D5	*ARPC3*	Highly similar to Homo sapiens actin-related protein 2/3 complex, subunit 3	2.45	1.73	0.92	0.39	0.48
	P59998	*ARPC4*	Actin-related protein 2/3 complex subunit 4	2.15	1.03	0.78	0.65	0.41
	B3KPC7	*ARPC5*	Actin-related protein 2/3 complex subunit 5	1.39	1.30	0.83	0.00	0.52

Actin-binding protein	P12814	*ACTN1*	Alpha-actinin-1	1.44	0.69	0.91	2.21	0.54
	B4DP09	*CNN3*	Highly similar to calponin-3	2.24	Inf	1.02	Und	0.79
	P23528	*CFL1*	Cofilin-1	1.72	0.70	0.58	1.08	0.88
	Q16643	*DBN1*	Drebrin	1.51	1.39	1.08	0.00	0.00
	P21333-2	*FLNA*	Isoform 2 of filamin-A	3.46	1.02	1.02	0.61	0.55
	O75369-2	*FLNB*	Isoform 2 of filamin-B	4.18	1.08	1.38	0.74	0.61

Regulator of actin dynamics	P62745	*RHOB*	RhoB	1.96	0.56	0.89	0.76	0.86
	Q5JR08	*RHOC*	RhoC	1.80	0.96	0.80	0.85	0.86
	P84095	*RHOG*	RhoG	1.30	0.78	5.60	0.74	0.40
	P63000	*RAC1*	Ras-related C3 botulinum toxin substrate 1	1.98	0.70	0.90	0.85	0.71
	P15153	*RAC2*	Ras-related C3 botulinum toxin substrate 2	1.87	0.74	1.30	0.73	0.83
	P62826	*RAN*	Ran	1.16	0.73	0.68	0.58	0.98
	P60953	*CDC42*	Cell division control protein 42	1.49	0.61	1.00	0.88	0.86

Small GTPase	P01112	*HRAS*	H-Ras	2.55	0.37	0.99	0.40	0.96
	P01116-2	*KRAS*	K-Ras	3.46	0.33	0.83	0.78	0.84
	P01111	*NRAS*	N-Ras	3.06	0.40	0.83	0.56	0.82
	P10301	*RRAS*	Ras-related protein R-Ras	2.36	0.98	1.00	0.34	1.01
	P62070	*RRAS2*	Ras-related protein R-Ras2	1.66	0.59	0.83	0.31	0.64

Guanine nucleotide exchange factor	Q86TW5	*ARHGEF16*	Rho guanine nucleotide exchange factor 16	2.96	0.99	1.10	1.17	1.04
	P18754	*RCC1*	Regulator of chromosome condensation	2.46	Inf	0.58	Inf	1.00

a Shown are individual results from 3 biological replicates for epithelial cells infected with C. albicans and 2 biological replicates from epithelial cells incubated with EGF. Values are the ratio of the amount of protein detected in cells exposed to C. albicans or EGF relative to unstimulated control cells. “Inf” (i.e., “infinite change”) indicates proteins that were undetectable in unstimulated control cells.

The endocytosis of C. albicans is mediated in part by the clathrin and cortactin internalization pathway (21). Consistent with this mechanism, the clathrin heavy and light chains, cortactin, and the phosphatidylinositol-binding clathrin assembly protein were found to associate with EGFR (Table 2). Activation of the clathrin pathway induces rearrangement of actin filaments, leading to the formation of pseudopods that engulf the fungus, leading to the formation of an endocytic vacuole (22). We found that EGFR associated with all seven members of the actin-related protein 2/3 (arp2/3) complex, including actin-related proteins 2 and 3 and actin-related protein complex subunits 1B, 2, 3, 4, and 5 (Table 2). These proteins play a key role in organizing actin filaments during clathrin-mediated endocytosis and the formation of pseudopods (23, 24).

EGFR was also associated with numerous actin-binding proteins, including α-actinin, calponin 3, cofilin-1, drebrin, and fascin (Table 2). The association of EGFR with filamins A and B, which link actin to membrane glycoproteins (25), suggests that these both may connect EGFR with actin and its associated proteins. Actin dynamics are known to be regulated by small GTPases (26), and we found that EGFR was associated with RhoA, RhoB, RhoC, RhoG, RAC1, RAC2, Ran, and CDC42 (Table 2). EGFR associated with additional small GTPases, including H-Ras, K-Ras, N-Ras, R-Ras, R-Ras2 and 28 different Rabs (Table 2). Most small GTPases require guanine nucleotide-exchange factors (GEFs) for activation (27), and EFGR associated with guanine nucleotide exchange factor 16 (neuroblastoma, ephexin 4) and regulator of chromosome condensation 1 (RCC1) (Table 2). Collectively, these results indicate that EGFR has extensive interactions with the actin cytoskeleton and its regulatory proteins that likely induce the endocytosis of C. albicans hyphae and secretion of proinflammatory mediators when EGFR is activated.

### The EGFR-associated proteins WBP2, TOLLIP, IFITM3, and gC1qR govern the epithelial cell response to C. albicans.

Among the proteins that were predicted by the proteomics data to have increased association with EGFR in response to C. albicans, we selected six for additional study: WBP2, guanylate binding protein 6 (GBP6), TOLLIP, early endosome antigen 1 (EEA1), desmoglein-3 (DSG3), and IFITM3. These proteins were chosen because of their potential roles in governing the endocytosis of C. albicans and the secretion of proinflammatory mediators. We also investigated the globular C1q receptor (gC1qR), which was constitutively associated with EGFR. Our rationale was that gC1qR is known to function as a receptor for Listeria monocytogenes, and there are significant similarities between the mechanisms of host cell invasion by this bacterium and by C. albicans (18, 21, 28–30).

**FIG 1:**
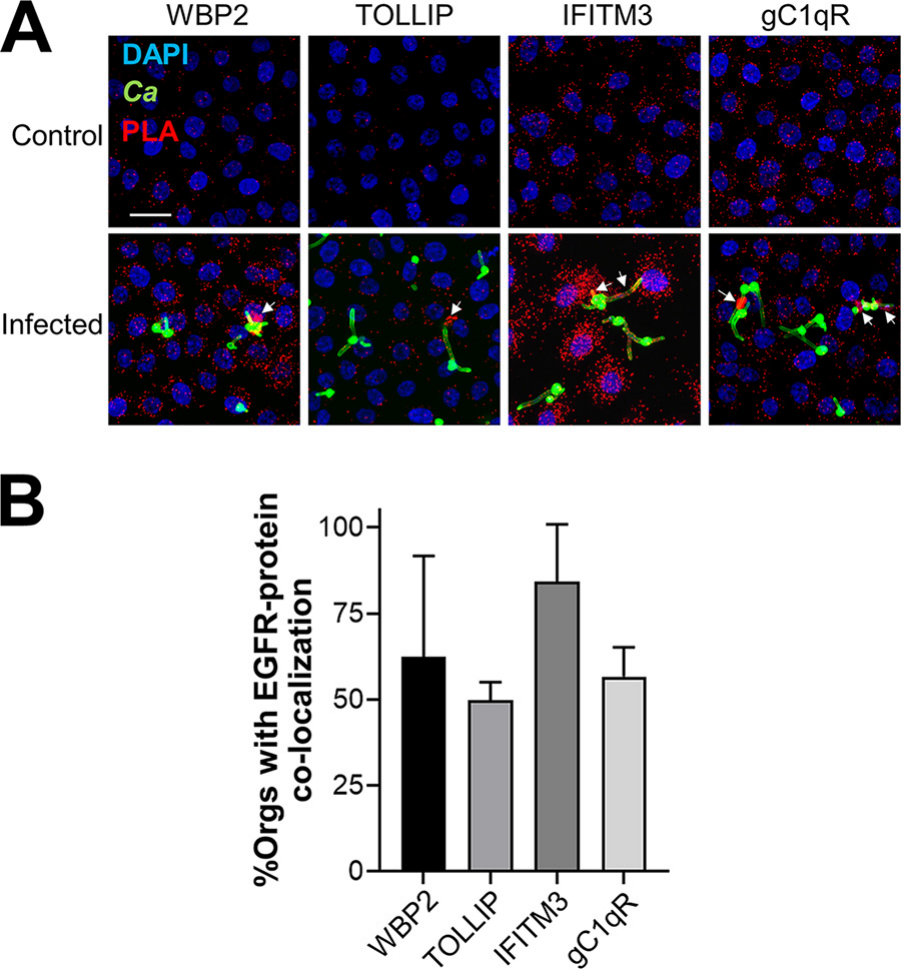
Proximity ligation assays (PLA) showing the physical association of the epidermal growth factor receptor (EGFR) with WW domain-binding protein 2 (WBP2), Toll-interacting protein (TOLLIP), interferon-induced transmembrane protein 3 (IFITM3), and the globular C1q receptor (gC1qR) in the OKF6/TERT-2 oral epithelial cell line. (A) Confocal microscopic images of epithelial cells that had been either incubated with medium alone (top) or infected with C. albicans (bottom) for 90 min. Red spots indicate the regions where the indicated proteins associate with EGFR. Arrows indicate the accumulation of the proteins around C. albicans hyphae. Results are representative of three independent experiments. Scale bar, 25 μm. (B) Quantitative analysis of the images to determine the percentage of C. albicans cells with spots indicating the colocalization of EGFR with WBP2, TOLLIP, IFITM3, or gC1qR. Results are the mean ± SD from three independent experiments. *Ca*, C. albicans; Orgs, organisms.

To verify that the selected proteins were associated with EGFR in intact oral epithelial cells and to determine their subcellular location with respect to C. albicans cells, we used a proximity ligation assay (16). This assay forms a fluorescent spot where two proteins are located within 40 nm of one another (31). All seven proteins were associated with EGFR in both infected and uninfected epithelial cells, but only WBP2, TOLLIP, IFITM3, and gC1qR accumulated with EGFR in the vicinity of C. albicans hyphae (Fig. 1; see Fig. S1 in the supplemental material). The accumulation of WBP2, TOLLIP, IFITM3, and gC1qR around C. albicans suggested that these proteins might govern the epithelial cell response to the fungus.

10.1128/mBio.02716-21.1 FIG S1 Proximity ligation assays (PLA) to assess the physical association of the epidermal growth factor receptor (EGFR) with guanylate binding protein 6 (GBP6), early endosome antigen 1 (EEA1), and desmoglein-3 (DSG3) in the OKF6/TERT-2 oral epithelial cell line. (A) Confocal microscopic images of epithelial cells incubated in either medium alone (top) or infected with C. albicans (bottom) for 90 min. Red spots indicate the regions where the indicated proteins associate with EGFR. Results are representative of three independent experiments. Scale bar, 25 μm. (B) Quantitative analysis of the images to determine the percentage of C. albicans cells with spots indicating the colocalization of EGFR with GPB2, EEA1, or DSG3. Results are the mean ± SD from three independent experiments. Orgs, organisms. Download FIG S1, PDF file, 0.3 MB. Copyright © 2021 Phan et al. 2021 Phan et al. https://creativecommons.org/licenses/by/4.0/ This content is distributed under the terms of the Creative Commons Attribution 4.0 International license.

**FIG 2:**
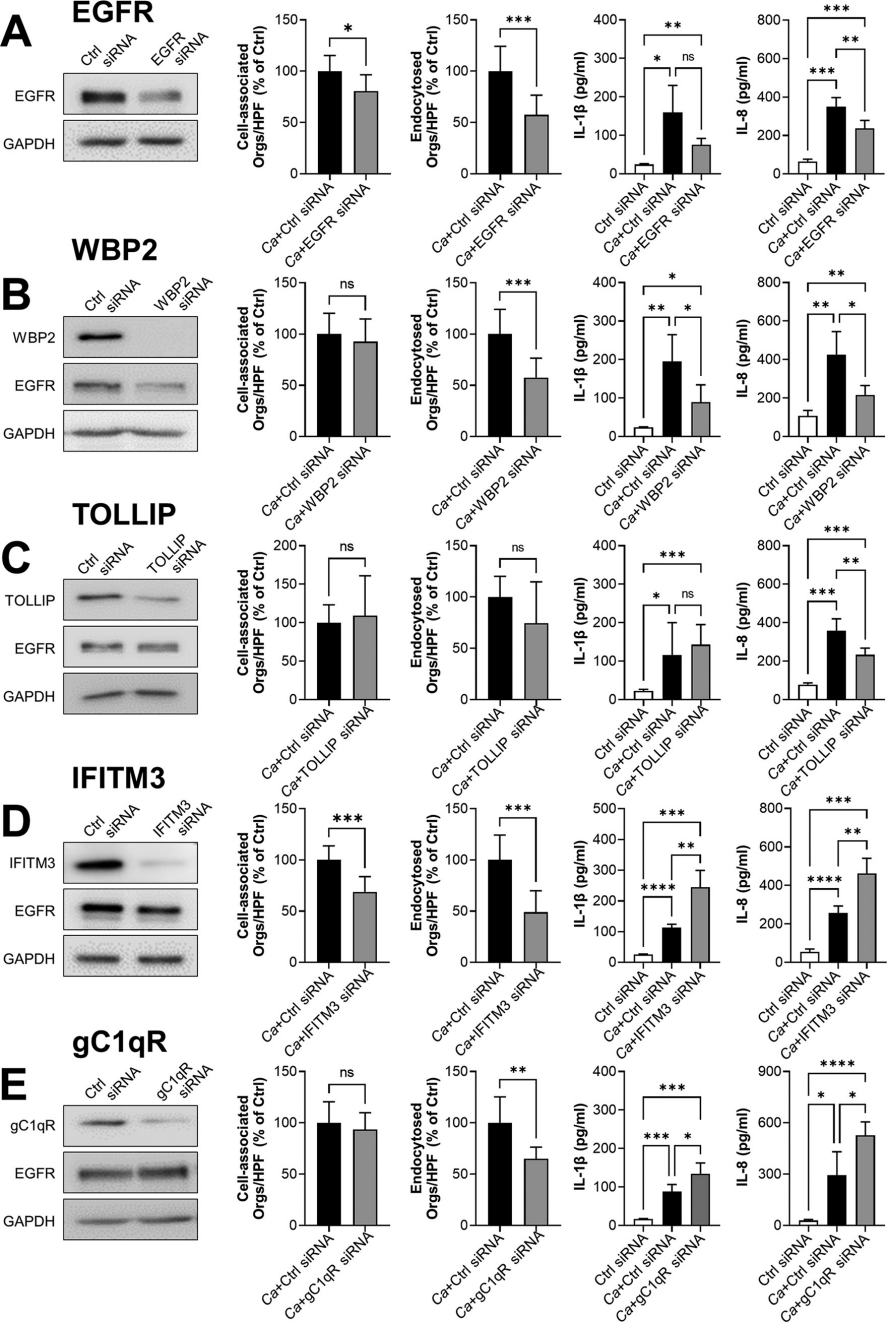
Functional analysis of proteins that interact with EGFR. Oral epithelial cells were transfected with EGFR (A), WBP2 (B), TOLLIP (C), IFITM3 (D), and gC1qR (E) siRNA. For each siRNA, the extent of protein knockdown and its effects on the number of cell-associated organisms, the number of endocytosed organisms, IL-1β secretion, and IL-8 secretion were determined. The graphs show the mean ± SD from three independent experiments, each performed in triplicate. The data were analyzed using one-way analysis of variance with Dunnett’s test for multiple comparisons. *Ca*, C. albicans; Ctrl, control; ns, not significant; Orgs/HPF, organisms per high-power field; *, *P* < 0.05; **, *P* < 0.01; ***, *P* < 0.001; ****, *P* < 0.0001.

To investigate this hypothesis, we used small interfering (siRNA) to knock down the levels of each these proteins in the OKF6/TERT-2 human oral epithelial cell line. We then measured C. albicans adherence to epithelial cells and subsequent endocytosis using a standard differential fluorescence assay (18, 22). We also analyzed the secretion of IL-1β and IL-8 by enzyme-linked immunosorbent assay (ELISA). As a control, we used siRNA to knock down EGFR in the epithelial cells. Consistent with previous results (13, 16), knockdown of EGFR modestly reduced the number of cell-associated C. albicans cells (a measure of adherence) and decreased the number of endocytosed organisms by almost 50% (Fig. 2A). Reduction of EGFR also inhibited C. albicans*-*induced secretion of IL-8 but not IL-1β.

WBP2 is a multifunctional protein that has mainly been studied in the context of breast cancer. Functioning as an adapter protein and transcriptional coactivator, WBP2 is required for maximal EGFR expression and for the normal activity of the phosphatidylinositol 3-kinase (PI3K)/Akt signaling pathway (32). WBP2 also links Jun N-terminal protein kinase (JNK) to Wnt signaling (32). Both PI3k/Akt and JNK have been shown to govern the response of oral epithelial cells to C. albicans (33, 34). As shown, siRNA knockdown of WBP2 reduced total EGFR levels in oral epithelial cells, leading to a decrease in C. albicans endocytosis and a reduction in IL-1β and IL-8 production (Fig. 2B). Thus, knockdown of WBP2 largely phenocopies the knockdown of EGFR and is required for normal cellular EGFR levels in oral epithelial cells.

TOLLIP is a membrane-associated endocytic adapter protein that is a negative regulator of the innate immune response. TOLLIP inhibits signaling by STAT1, Toll-like receptors, and IL-1β (35–37), but was not previously known to associate with EGFR. Knockdown of TOLLIP inhibited secretion of IL-8 but had no impact on the adherence or endocytosis of C. albicans or the secretion of IL-1β (Fig. 2C). These results suggest that TOLLIP is a positive regulator of epithelial cell IL-8 secretion in response to C. albicans infection.

IFITM3 plays a key role in the host defense against viral infections by binding to virus particles and shuttling them to lysosomes for degradation (38). By a similar process, IFITM3 also enhances the degradation of activated EGFR (38). Knockdown of IFITM3 in oral epithelial cells had paradoxical effects. Although loss of IFITM3 inhibited the adherence and endocytosis of C. albicans, it stimulated C. albicans-induced secretion of IL-1β and IL-8 (Fig. 2D), thereby dissociating the process of endocytosis from cytokine production.

gC1qR (HABP1/p32) is present in the mitochondrial matrix where is involved in oxidative phosphorylation (39). Although gC1qR lacks a transmembrane sequence, it is also expressed on the cell surface, where it functions as a receptor for C1q, high-molecular weight kininogen, factor XII, vitronectin, and hyaluronic acid (40–43). gC1qR also acts as a host cell receptor for multiple pathogens, including L. monocytogenes, Staphylococcus aureus, and Plasmodium falciparum (28, 44, 45). In carcinoma cells, inhibition of gC1qR with a monoclonal antibody is known to reduce EGFR phosphorylation and block stimulation of migration and formation of lamellipodia in response to EGF (46). We found that siRNA knockdown of gC1qR inhibited the endocytosis of C. albicans but stimulated the secretion of IL-1β and IL-8 (Fig. 2E), suggesting that gC1qR may play a key role in the activation of EGFR by C. albicans.

### Surface-exposed gC1qR is required for EGFR-mediated endocytosis of C. albicans.

**FIG 3:**
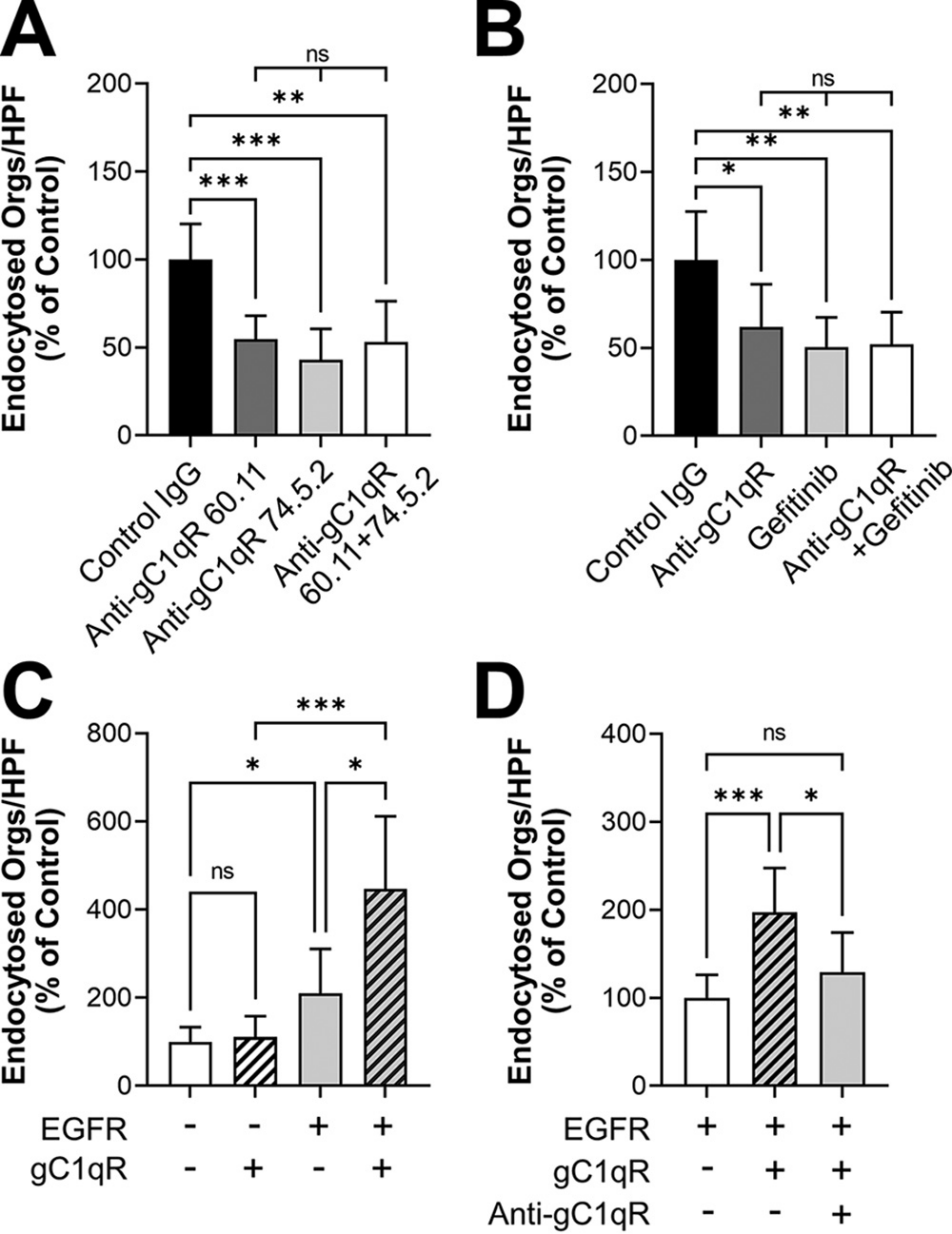
Surface-expressed gC1qR mediates the endocytosis of C. albicans. (A) Effects of two different anti-gC1qR monoclonal antibodies on the endocytosis of C. albicans by oral epithelial cells. (B) Effects of the anti-gC1qR antibody 74.5.2 and the EGFR kinase inhibitor gefitinib on the endocytosis of C. albicans by oral epithelial cells. (C and D) Endocytosis of C. albicans by NIH/3T3 cells expressing human gC1qR and/or human EGFR. (C) Additive effects of EGFR and gC1qR on endocytosis. (D) Effects of inhibiting surface-expressed gC1qR with the anti-gC1qR antibody 74.5.2 on endocytosis. Results are the mean ± SD from three independent experiments, each performed in triplicate. The data were analyzed using one-way analysis of variance with Dunnett’s test for multiple comparisons. ns, not significant; Orgs/HPF, organisms per high-power field; *, *P* < 0.05; **, *P* < 0.01; ***, *P* < 0.001.

Because gC1qR functions as a host cell receptor for several microbial pathogens and was required for the maximal endocytosis of C. albicans, we focused on this protein for in-depth study. gC1qR is expressed both on the cell surface and intracellularly. Thus, the gC1qR siRNA likely reduced the levels of both cell surface and intracellular gC1qR. To test whether the inhibition of just surface-exposed gC1qR altered the epithelial cell response to C. albicans, we evaluated two different anti-gC1qR monoclonal antibodies, 60.11 and 74.5.2, which bind to different domains of gC1qR (47). Both antibodies decreased the endocytosis of C. albicans (Fig. 3A), and antibody 74.5.2 slightly reduced the number of cell-associated organisms (see Fig. S2A in the supplemental material). Also, treating the epithelial cells with both monoclonal antibodies together did not result in a further inhibition of endocytosis. Thus, surface-exposed gC1qR is required for maximal endocytosis of C. albicans by oral epithelial cells.

10.1128/mBio.02716-21.2 FIG S2 Surface-expressed gC1qR has minimal effects on the number of C. albicans cells that are associated with oral epithelial cells. (A) Effects of two different anti-gC1qR monoclonal antibodies on the number of cell-associated C. albicans cells. (B) Effects of the anti-gC1qR antibody 74.5.2 and the EGFR kinase inhibitor gefitinib on the number of cell-associated C. albicans cells. (C and D) Number of C. albicans cells that are cell associated with NIH/3T3 cells expressing human gC1qR and/or human EGFR. (C) Effects of EGFR and gC1qR expression on cell association. (D) Effects of inhibiting surface-expressed gC1qR with the anti-gC1qR antibody 74.5.2. Results are the mean ± SD from three independent experiments, each performed in triplicate. The data were analyzed using one-way analysis of variance with Dunnett’s test for multiple comparisons. ns, not significant; Orgs/HPF, organisms per high-power field; *, *P* < 0.05. Download FIG S2, PDF file, 0.3 MB. Copyright © 2021 Phan et al. 2021 Phan et al. https://creativecommons.org/licenses/by/4.0/ This content is distributed under the terms of the Creative Commons Attribution 4.0 International license.

To investigate the functional relationship between gC1qR and EGFR, we treated the oral epithelial cells with an anti-gC1qR monoclonal antibody, the specific EGFR inhibitor gefitinib, or the antibody and gefitinib in combination. Both the anti-gC1qR antibody and gefitinib inhibited the endocytosis of C. albicans similarly, and combining the anti-gC1qR antibody with gefitinib did not decrease endocytosis further (Fig. 3B). None of the treatments altered the number of cell-associated organisms (Fig. S2B). These results suggest that gC1qR may function in the same pathway as EGFR to mediate the endocytosis of C. albicans.

We further explored the relationship between gC1qR and EGFR in the endocytosis of C. albicans using a heterologous expression approach. We obtained two NIH/3T3 mouse fibroblastoid cell lines: a wild-type cell line and one that had been transfected with human EGFR and HER2 (48). Each of these cell lines was then transfected with either green fluorescent protein (GFP) as a control or human gC1qR. When wild-type NIH/3T3 cells were transfected with gC1qR, they endocytosed a similar number of C. albicans cells to the control cells (Fig. 3C), indicating that gC1qR was unable to induce endocytosis in the absence of EGFR. As we found previously (13), NIH/3T3 cells that expressed human EGFR and HER2 endocytosed more C. albicans cells than the wild-type cells. When the EGFR-expressing cells were transfected with gC1qR, they endocytosed even more organisms. This increase in endocytosis was due to the presence of surface-expressed gC1qR because treating these cells with an anti-gC1qR antibody reduced endocytosis to basal levels (Fig. 3D). There was an increase in the number of C. albicans cells that were associated with NIH/3T3 cells that expressed EGFR relative to cells that did not (Fig. S2C). However, expression of gC1qR had no significant effect on the number of cell-associated organisms (Fig. S2C and D). Collectively, these data indicate that gC1qR is a key cofactor that enhances EGFR-mediated endocytosis of C. albicans.

### Surface-expressed gC1qR is required for C. albicans*-*induced stimulation of epithelial cell production of proinflammatory mediators.

**FIG 4:**
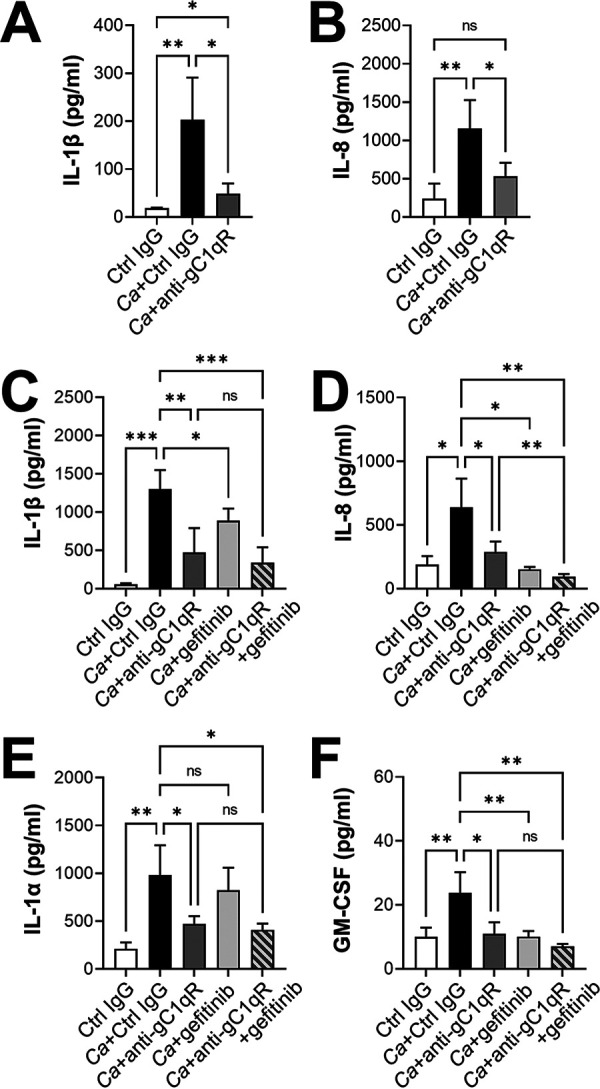
gC1qR is required for production of proinflammatory mediators by oral epithelial cells in response to C. albicans infection. (A to F) Oral epithelial cells were infected with C. albicans in the presence of the anti-gC1qR antibody 74.5.2 or gefitinib for 8 h, after which the concentration of the indicated inflammatory mediators in the medium was analyzed by ELISA (A and B) or Luminex cytometric bead array (C to F). Results are the mean ± SD from three independent experiments, each performed in duplicate. The data were analyzed using one-way analysis of variance with Dunnett’s test for multiple comparisons. *Ca*, C. albicans; Ctrl, control; ns, not significant; *, *P* < 0.05; **, *P* < 0.01; ***, *P* < 0.001.

To investigate the role of surface-expressed gC1qR in the oral epithelial cell inflammatory response to C. albicans, we analyzed the effects of an anti-gC1qR antibody on the secretion of IL-1β and IL-8. In contrast to knockdown of gC1qR with siRNA, inhibiting gC1qR with the monoclonal antibody significantly reduced the secretion of both mediators, indicating that surface-expressed gC1qR is required for C. albicans to stimulate their production (Fig. 4A and B).

Next, we analyzed the functional relationship between gC1qR and EGFR in the epithelial cell proinflammatory response. We used a cytometric bead array to measure the levels of IL-1β, IL-8, IL-1α, and granulocyte-macrophage colony-stimulating factor (GM-CSF) that were secreted in response to C. albicans. Inhibition of surface-expressed gC1qR significantly reduced the production of all four inflammatory mediators (Fig. 4C to F), although the absolute levels of IL-1β and IL-8 were somewhat different when measured by the cytometric bead array instead of the ELISA. Inhibition of EGFR with gefitinib also significantly decreased the levels of IL-1β, IL-8, and GM-CSF, but had no effect on IL-1α levels, as reported previously (15, 16). The finding that inhibition of gC1qR reduced IL-1α production whereas inhibition of EGFR had no effect suggests that gC1qR is required for the production of IL-α by interacting with a receptor other than EGFR.

When the epithelial cells were incubated with the anti-gC1qR antibody and gefitinib in combination, the production of IL-1β and GM-CSF was not inhibited more than in cells incubated with the anti-gC1qR antibody alone (Fig. 4C and F). However, dual inhibition of gC1qR and EGFR resulted in a modest further reduction in IL-8 production (Fig. 4F). Collectively, these results indicate that gC1qR and EGFR function in the same pathway to induce the production of IL-1β, IL-8, and GM-CSF in response to C. albicans.

### gC1qR is necessary for intact C. albicans to interact with EGFR.

**FIG 5:**
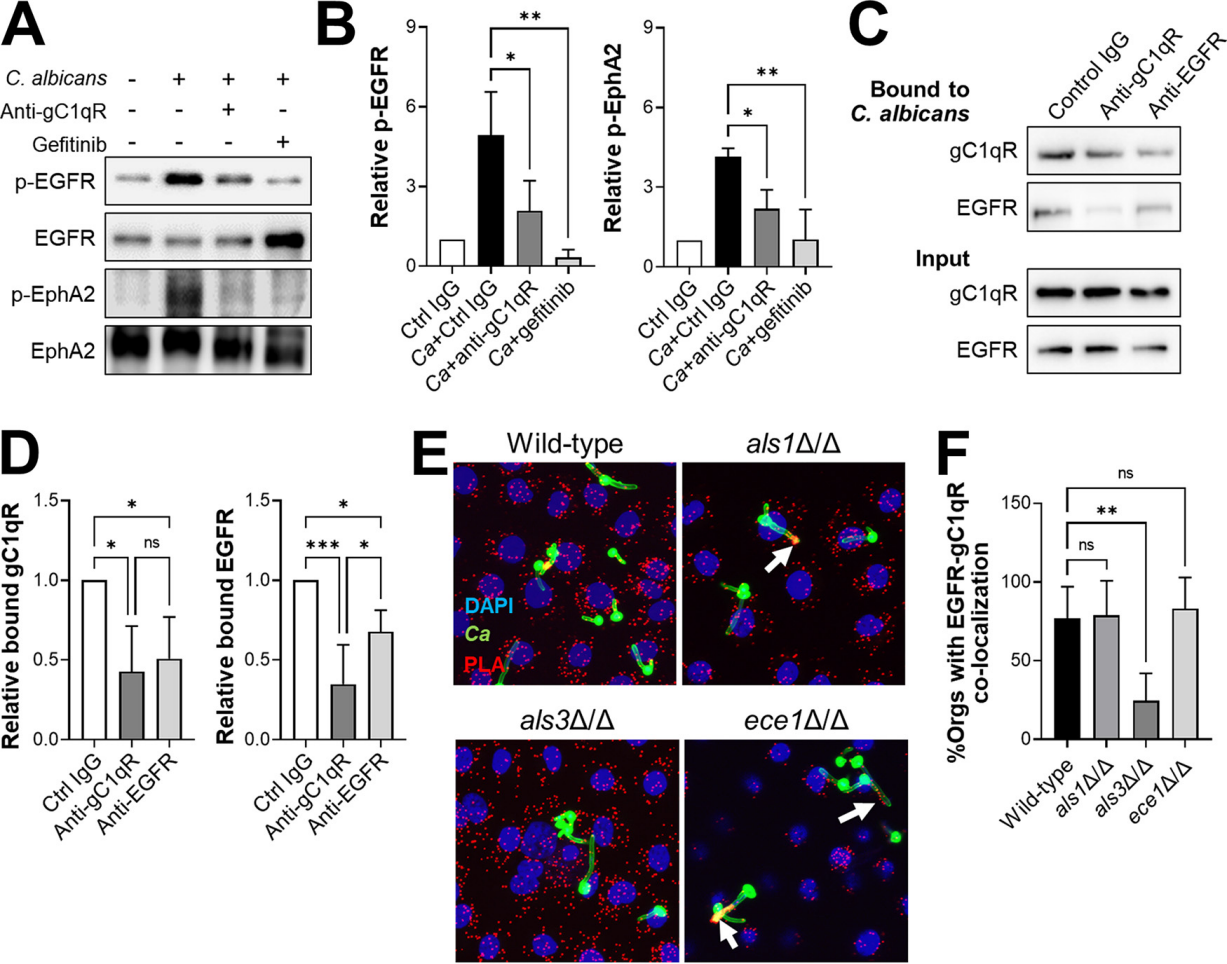
Interactions of gC1qR and EGFR with C. albicans. (A and B) Effects of the anti-gC1qR antibody 74.5.2 and gefitinib on the phosphorylation of EGFR and EphA2 in response to 90-min infection with C. albicans. (A) Representative immunoblots. (B) Densitometric analysis of three immunoblots, such as the ones shown in panel A. (C and D) Effects of anti-gC1qR and anti-EGFR antibodies on binding of gC1qR and EGFR to C. albicans hyphae. (C) Representative immunoblots. (D) Densitometric analysis of four immunoblots, such as the ones shown in panel C. Results are the mean ± SD from 3 to 4 independent experiments. (E and F) Representative confocal images of the proximity ligation assay (PLA) showing the association of gC1qR with EGFR around hyphae of C. albicans wild-type, *als1*Δ/Δ, and *ece1*Δ/Δ strains, but not the *als3*Δ/Δ mutant (E). (F) Quantitative analysis of the images such as the one in panel E to determine the percentage of hyphae of the indicated C. albicans strains, with spots indicating the colocalization of EGFR with gC1qR. Results are the mean ± SD from three independent experiments. The numerical data were analyzed using one-way analysis of variance with Dunnett’s test for multiple comparisons. *Ca*, C. albicans; Ctrl, control; ns, not significant; Orgs, organisms; *, *P* < 0.05; **, *P* < 0.01; ***, *P* < 0.001.

Our finding that gC1qR functions in the same pathway as EGFR to mediate epithelial cell endocytosis and stimulation prompted us to investigate whether gC1qR was necessary for C. albicans to activate EGFR. We found that treatment of oral epithelial cells with an anti-gC1qR antibody decreased C. albicans-induced phosphorylation of EGFR by approximately 58% (Fig. 5A and C). As expected, treatment of the infected cells with the EGFR kinase inhibitor gefitinib reduced EGFR phosphorylation to below basal levels. Notably, inhibition of gC1qR did not block EGFR phosphorylation in response to either EGF or candidalysin, a pore-forming toxin released by C. albicans (15, 49) (see Fig. S3 in the supplemental material). Thus, the effects of inhibiting surface-expressed gC1qR were specific to intact C. albicans.

10.1128/mBio.02716-21.3 FIG S3 Inhibition of gC1qR does not block EGFR phosphorylation in response to 40 μM candidalysin (A) or 1 ng/ml epidermal growth factor (EGF) (B). Representative Western blots from three independent experiments. Download FIG S3, PDF file, 0.02 MB. Copyright © 2021 Phan et al. 2021 Phan et al. https://creativecommons.org/licenses/by/4.0/ This content is distributed under the terms of the Creative Commons Attribution 4.0 International license.

Activation of EGFR is also required for C. albicans to induce sustained phosphorylation of the EphA2 receptor tyrosine kinase (11, 16). We determined that inhibition of gC1qR decreased C. albicans-induced EphA2 phosphorylation (Fig. 5A and B). These findings are consistent with the model that gC1qR is necessary for C. albicans to activate EGFR and its downstream signaling pathways in oral epithelial cells.

Previously, we found that EGFR associated, either directly or indirectly, with C. albicans hyphae (13). To determine if gC1qR plays a role in this association, we infected oral epithelial cells with C. albicans in the presence of either an anti-gC1qR antibody or an anti-EGFR antibody. After 90 min, we lysed the epithelial cells with a detergent, collected the C. albicans hyphae, and rinsed them extensively to remove unbound proteins. Using high-molar urea, we eluted the epithelial cell proteins that remained associated with the organisms and analyzed them by immunoblotting. In control cells, both gC1qR and EGFR were associated with the C. albicans hyphae (Fig. 5C and D). When the cells were incubated with the anti-gC1qR antibody, there was a significant reduction in the amounts of gC1qR and EGFR that were associated with C. albicans. When the cells were incubated with an anti-EGFR antibody, the amounts of fungus-associated gC1qR and EGFR were also significantly reduced. Collectively, these data suggest that gC1qR and EGFR have a reciprocal relationship such that each protein is necessary for the other to maximally associate with C. albicans.

### C. albicans Als3 but not candidalysin is required for increased association of gC1qR with EGFR.

Both the Als3 invasin and the candidalysin pore-forming toxin are required for C. albicans to maximally activate EGFR in oral epithelial cells (15, 16). We investigated whether these factors were required for gC1qR to associate with EGFR around C. albicans cells in intact oral epithelial cells. Using the proximity ligation assay, we determined that when oral epithelial cells were infected with an *als3*Δ/Δ deletion mutant, there was minimal accumulation of the gC1qR-EGFR-containing complex around the hyphae (Fig. 5E and F). In contrast, when the epithelial cells were infected with the wild-type strain, an *als1*Δ/Δ deletion mutant, or a candidalysin-deficient *ece1*Δ/Δ deletion mutant, this complex formed around the hyphae. Thus, Als3, but not Als1 or candidalysin, is necessary for gC1qR to associate with EGFR around C. albicans hyphae.

### Inhibition of gC1qR does not alter C. albicans virulence or activation of EGFR in mice.

**FIG 6:**
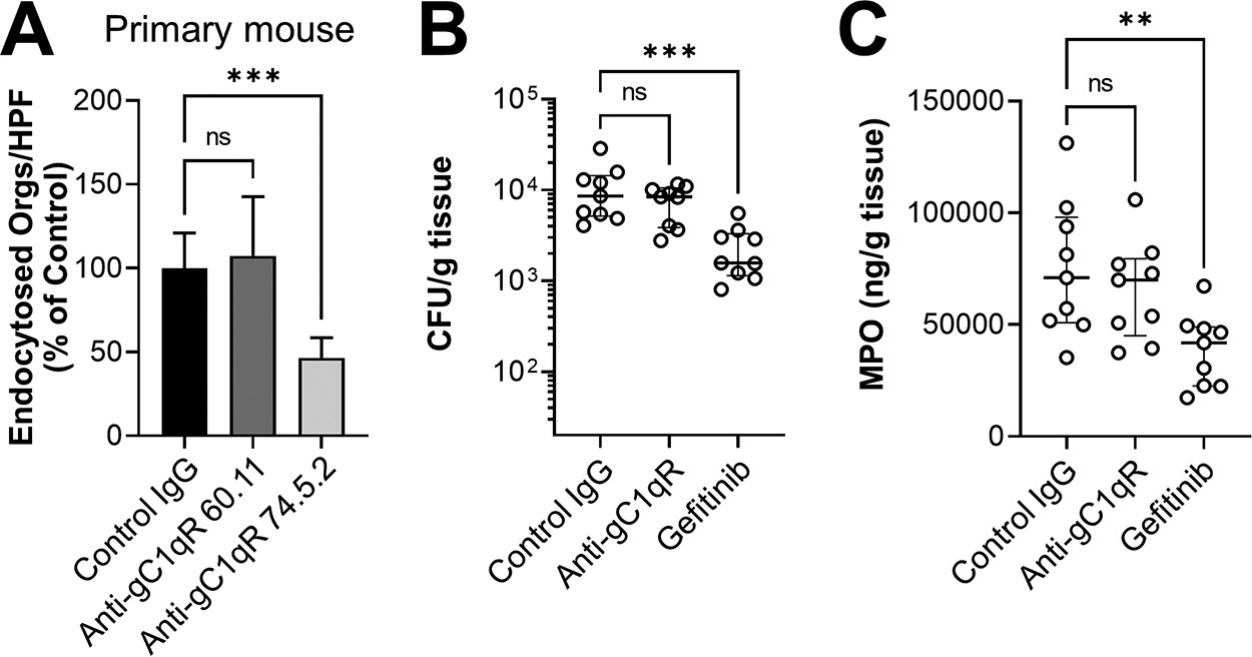
Inhibition of gC1qR has no significant effect on the outcome of oropharyngeal candidiasis. (A) Effects of the indicated anti-gC1qR antibodies on the endocytosis of C. albicans by primary mouse oral epithelial cells. Results are the mean ± SD from three independent experiments, each performed in triplicate. The data were analyzed using one-way analysis of variance with Dunnett’s test for multiple comparisons. (B and C) Effects of the anti-gC1qR antibody 74.5.2 and gefitinib on the outcome of oropharyngeal candidiasis after 1 day of infection as shown by oral fungal burden (B) and oral myeloperoxidase (MPO) content (C). Results are the median ± interquartile range from two independent experiments, each with 4 to 5 mice per group. The data were analyzed using the Kruskal-Wallis test. ns, not significant; Orgs/HPF, organisms per high-power field; **, *P* < 0.01; ***, *P* < 0.001.

Next, we investigated the role of gC1qR in the pathogenesis of oropharyngeal candidiasis in mice. To verify that monoclonal antibodies raised against human gC1qR could inhibit mouse gC1qR, we tested the capacity of these antibodies to inhibit the endocytosis of C. albicans by primary mouse epithelial cells. We found monoclonal antibody 74.5.2, which is directed against the binding site for high-molecular-weight kininogen (47), significantly reduced C. albicans endocytosis (Fig. 6A). It also decreased the number of cell-associated organisms (see Fig. S4 in the supplemental material). In contrast, monoclonal antibody 60.11, which recognizes the binding site for C1q (47), had no detectable effect on either interaction.

10.1128/mBio.02716-21.4 FIG S4 Effects of anti-gC1qR antibodies and gC1qR siRNA on the interactions of C. albicans with human oral epithelial cells and primary mouse oral epithelial cells. (A and B) Effects of the indicated anti-gC1qR antibodies on adherence to primary mouse (A) and primary human (B) oral epithelial cells. (C-E) Effects of siRNA knockdown of gC1qR on the phosphorylation of EGFR in primary mouse oral epithelial cells. (C) Representative Western blot. (D) Densitometric analysis of four Western blots, such as the one shown in panel C. Results in panels A and B are the mean ± SD from three independent experiments, each performed in triplicate. The data were analyzed using one-way analysis of variance with Dunnett’s test for multiple comparisons. *Ca*, C. albicans; Ctrl, control; ns, not significant; Orgs/HPF, organisms per high-power field; *, *P* < 0.05; **, *P* < 0.01. Download FIG S4, PDF file, 0.3 MB. Copyright © 2021 Phan et al. 2021 Phan et al. https://creativecommons.org/licenses/by/4.0/ This content is distributed under the terms of the Creative Commons Attribution 4.0 International license.

Based on these results, we treated immunocompetent mice with antibody 74.5.2 and orally infected them with wild-type C. albicans. Control mice were treated with either mouse IgG or gefitinib. We found that after 1 day of infection, mice treated with the anti-gC1qR antibody had the same oral fungal burden as mice that received the control IgG (Fig. 6B). The level of myeloperoxidase (MPO), a measure of phagocyte accumulation (50, 51), in the oral tissues was also not changed by administration of the anti-gC1qR antibody (Fig. 6C). As expected (16), treatment with gefitinib significantly reduced both the oral fungal burden and oral MPO levels in the mice. These results suggest the gC1qR is dispensable for mediating the epithelial cell response to C. albicans in mice.

### Human oral epithelial cells respond differently to C. albicans than mouse oral epithelial cells.

**FIG 7:**
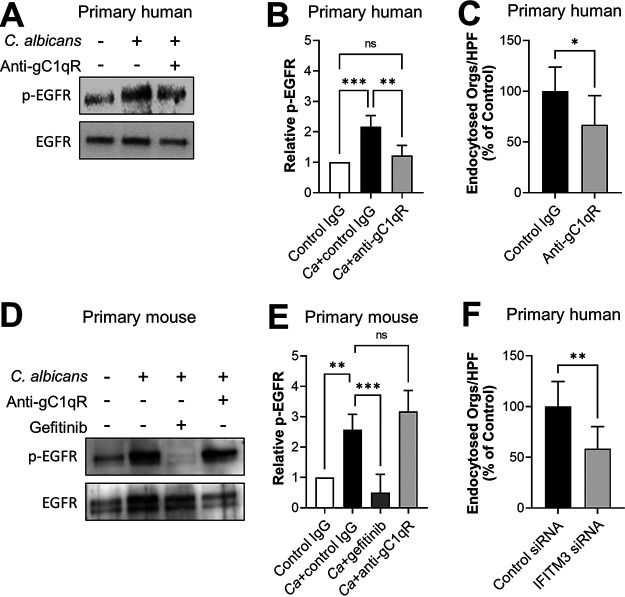
Mouse and human oral epithelial cells respond differently to C. albicans. (A and B) Effects of the anti-gC1qR antibody 74.5.2 on the phosphorylation of EGFR in primary human oral epithelial cells in response to 90-min infection with C. albicans. (A) Representative immunoblots. (B) Densitometric analysis of four immunoblots, such as the ones shown in panel A. (C) The anti-gC1qR antibody 74.5.2 inhibits the endocytosis of C. albicans by primary human oral epithelial cells. (D and E) Effects of the anti-gC1qR antibody 74.5.2 and gefitinib on the phosphorylation of EGFR in primary mouse oral epithelial cells in response to 90-min infection with C. albicans. (D) Representative Western blots. (E) Densitometric analysis of four Western blots, such as the ones shown in panel D. (F) Effects of siRNA knockdown of IFITM3 on primary human oral epithelial cells. Results are the mean ± SD from three independent experiments, each performed in triplicate. The data were analyzed using one-way analysis of variance with Dunnett’s test for multiple comparisons. *Ca*, C. albicans; ns, not significant; Orgs/HPF, organisms per high-power field; *, *P* < 0.05; **, *P* < 0.01; ***, *P* < 0.001.

One potential explanation for the lack of efficacy of the anti-gC1qR antibody in mice is that EGFR signaling pathways differ between mouse and human oral epithelial cells. To investigate this possibility, we analyzed the effects of knocking down or inhibiting both gC1qR and IFITM3 in human and mouse oral epithelial cells. Because our original experiments were performed with the human OKF6/TERT-2 oral epithelial cell line, we first verified that primary human oral epithelial cells responded similarly to this cell line. We found that treatment of primary human oral epithelial cells with the anti-gC1qR antibody reduced C. albicans-induced phosphorylation of EGFR and C. albicans endocytosis (Fig. 7A to C) but had a nonsignificant effect on adherence (Fig. S4B), similar to its activity on the OKF6/TERT-2 cell line (Fig. 3A and Fig. 5A and B). Thus, gC1qR is necessary for C. albicans to activate EGFR and induce the endocytosis of C. albicans by human oral epithelial cells.

Next, we tested the capacity of the anti-gC1qR antibody 74.5.2 to inhibit C. albicans*-*induced phosphorylation of EGFR in primary mouse epithelial cells. We determined that the antibody had no effect, although EGFR phosphorylation was inhibited by gefitinib (Fig. 7D and E). Knockdown of gC1qR in mouse epithelial cells also failed to reduce EGFR phosphorylation in responses to C. albicans (Fig. S4C and D). Collectively, these data indicate that in mice, gC1qR is likely dispensable for C. albicans-induced EGFR activation, unlike in humans.

These results prompted us to investigate whether IFITM3 also functioned differently in human versus mouse oral epithelial cells. In OKF6/TERT-2 cells, knockdown of IFITM3 did not decrease the levels of total or phosphorylated EGFR, so we did not investigate this response further (see Fig. S5A and B in the supplemental material). In primary human oral epithelial cells, siRNA knockdown of IFITM3 inhibited the endocytosis of C. albicans (Fig. 7F), similarly to its effects on the human OKF6/TERT-2 oral epithelial cell line (Fig. 2D). However, knockdown of IFITM3 in primary human epithelial cells did not inhibit adherence (Fig. S5C). In mouse oral epithelial cells, knockdown of IFITM3 had no effect on the endocytosis or adherence of C. albicans (Fig. S5D and E). These results suggest that gC1qR and IFITM3 function differently in human oral epithelial cells than in mouse oral epithelial cells.

10.1128/mBio.02716-21.5 FIG S5 Effects of siRNA knockdown of IFITM3 on the response of human and mouse oral epithelial cells to C. albicans. (A and B) Phosphorylation of EGFR in the human OKF5/TERT-2 oral epithelial cell line. (A) Representative Western blot. (B) Densitometric analysis of four Western blots, such as the one shown in panel A. (C-E) Effects of siRNA knockdown of IFITM3 on the adherence of C. albicans to primary human oral epithelial cells (C), endocytosis by primary mouse oral epithelial cells (D), and adherence to primary mouse oral epithelial cells (E). Results in panels C to E are the mean ± SD from three independent experiments, each performed in triplicate. The data were analyzed using one-way analysis of variance with Dunnett’s test for multiple comparisons. *Ca*, C. albicans; Ctrl, control; ns, not significant; Orgs/HPF, organisms per high-power field; *, *P* < 0.05; **, *P* < 0.01. Download FIG S5, PDF file, 0.3 MB. Copyright © 2021 Phan et al. 2021 Phan et al. https://creativecommons.org/licenses/by/4.0/ This content is distributed under the terms of the Creative Commons Attribution 4.0 International license.

## DISCUSSION

In this work, we determined that EGFR interacts with numerous proteins that function in a multitude of different signaling pathways, consistent with the concept that this receptor is a key regulator of epithelial cell physiology. The proteomics data indicate that EGFR is part of a multiprotein complex that contains E-cadherin, EphA2, and HER2. This result provides an explanation for our previous findings that these three receptors function with EGFR in the same pathway to induce the epithelial cell response to C. albicans (11, 13, 18).

Previously, we had determined that C. albicans activates the aryl hydrocarbon receptor, leading to the derepression of Src family kinases that phosphorylate EGFR (14). However, the member(s) of the Src family responsible for this phosphorylation remained unknown. The current finding that Lyn and Yes associate with EGFR suggests that these two members of the Src family kinases phosphorylate EGFR in response to C. albicans infection.

EGFR mediates the endocytosis of C. albicans by activating the cortactin-clathrin pathway, leading to the formation of pseudopods that engulf the organism and pull it into the epithelial cell (21, 22). The proteomics data were consistent with this mechanism and suggest that the arp2/3 complex mediates the rearrangement of actin filaments that induce pseudopod formation. Although the arp2/3 complex can be activated by the Wiskott-Aldrich syndrome protein (WASP) or vasodilator-stimulated phosphoprotein (VASP) (52–54), neither protein was consistently associated with EGFR in cells infected with C. albicans. Thus, it remains to be determined how EGFR activates this complex.

We also established roles for four key proteins that interact with EGFR and induce the epithelial cell response to C. albicans, none of which had been previously implicated in the response to fungal pathogens. Three of these proteins, WBP2, IFITM3, and gC1qR, were required for maximal epithelial cell endocytosis of the fungus, suggesting that they function along with EGFR to orchestrate this process.

Perhaps not surprisingly, siRNA knockdown of these proteins had pleiotropic effects on the production of IL-1β and IL-8 by the infected oral epithelial cells, as these are regulated by a myriad of inflammatory and infectious signals. Knockdown of WBP2 led to reduced cellular levels of EGFR and thus resulted in decreased production of IL-1β and IL-8, similar to siRNA knockdown of EGFR itself. This result is consistent with a report that WBP2 is required for normal EGFR expression (32).

Knockdown of TOLLIP inhibited the production of IL-8 but had no effect on IL-1β secretion. Although TOLLIP is generally considered to be a negative regulator of the host inflammatory response (35–37), our results suggest that it has the capacity to be a positive regulator of IL-8 production in epithelial cells infected with C. albicans.

While siRNA knockdown of IFITM3 inhibited C. albicans endocytosis, it stimulated the production of both IL-1β and IL-8. IFITM3 has been found to enhance the degradation of activated EGFR in pulmonary epithelial cells (38). Although knockdown of IFITM3 in oral epithelial cells had no detectable effect on total cellular EGFR levels or on EGFR phosphorylation, our results indicate that IFITM3 is a positive regulator of epithelial cell endocytosis of C. albicans but a negative regulator of cytokine production.

**FIG 8:**
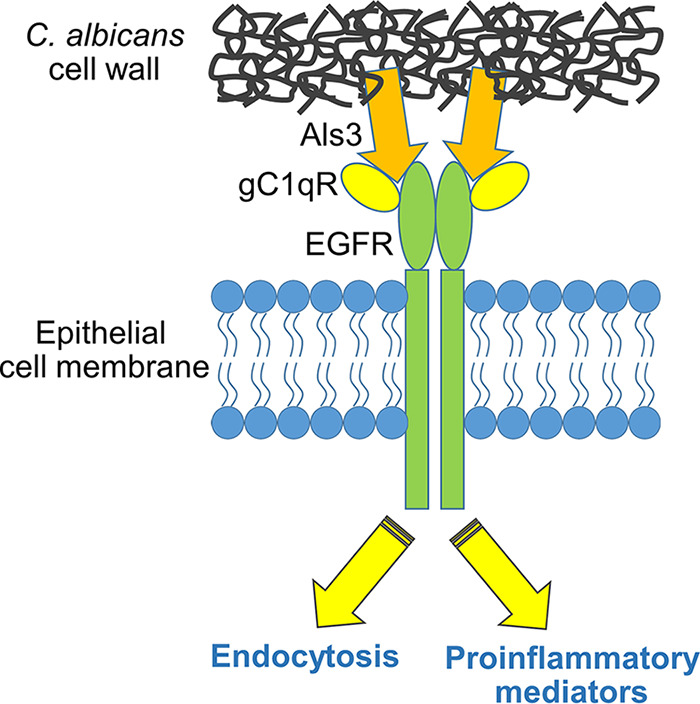
Diagram of the functional interaction of C. albicans Als3 with gC1qR and EGFR in human oral epithelial cells. Als3 interacts either directly or indirectly with both gC1qR and EGFR, leading to the activation of EGFR and subsequent induction of endocytosis and secretion of proinflammatory mediators.

Our in-depth analysis of gC1qR enabled us to develop a model for how this protein interacts with EGFR and is required for EGFR signaling in response to C. albicans infection (Fig. 8). We propose that gC1qR and EGFR form part of a complex that interacts either directly or indirectly with the C. albicans Als3 invasin. According to this model, gC1qR is required for Als3 to maximally interact with and activate EGFR. In turn, EGFR is required for C. albicans to interact optimally with gC1qR. This arrangement is deduced from the proteomics analysis, which showed that gC1qR was associated with EGFR, and from pulldown assays, which indicated that blocking gC1qR inhibited the physical association of EGFR with C. albicans and that blocking EGFR in turn reduced the association of gC1qR with the organism. Although the proteomics analysis suggested that gC1qR associated with EGFR constitutively, the proximity ligation assay showed that the gC1qR-EGFR complex was enriched in regions surrounding C. albicans hyphae that expressed Als3. Previously, we found that Als3 is required for C. albicans to induce maximal EGFR activation (13). Accordingly, these results suggest that gC1qR is a key cofactor that is required for Als3 to activate EGFR (Fig. 8).

Although EGF also activates EGFR, we found that it does so independently of gC1qR. These findings differ from those of Kim et al. (46), who reported that inhibition of gC1qR in the A549 lung cancer cell line blocked activation of EGFR induced by EGF. Possible reasons for these discrepant results include differences in the cell lines and the anti-gC1qR antibodies that we used compared to those used by the other investigators. We also found that blocking gC1qR did not prevent activation of EGFR by candidalysin. Collectively, these data suggest that soluble stimuli activate EGFR in oral epithelial cells independently of gC1qR, whereas C. albicans hyphae that express Als3 activate EGFR by a mechanism that requires gC1qR.

When investigating the role of gC1qR in mediating the production of IL-1β and IL-8 in response to C. albicans infection, we observed that treatment with the anti-gC1qR antibody inhibited the production of these inflammatory mediators, whereas siRNA knockdown of gC1qR had the opposite effect. The likely explanation for these results is that inhibiting just extracellular gC1qR has a different effect than decreasing the levels of intracellular gC1qR. Our experiments with the anti-gC1qR antibody indicate that it inhibits the production of proinflammatory mediators by blocking C. albicans-induced activation of EGFR and EphA2. These latter two receptors play key roles in stimulating the production of cytokines and chemokines in response to C. albicans (15, 16). Why reducing the levels of intracellular gC1qR stimulates the production of proinflammatory mediators in oral epithelial cells is unknown. However, knockdown of gC1qR in macrophages is known to reduce the cytoplasmic levels of tumor necrosis factor alpha-inducible protein 3 (TNFAIP3, A20), a suppressor of NF-κB activation, thereby stimulating the production of proinflammatory mediators (55). We speculate that a similar process may occur when gC1qR is knocked down in oral epithelial cells.

Inhibition of EGFR in mice with oropharyngeal candidiasis reduces oral fungal burden and inflammation (16). Thus, we were surprised to determine that treating mice with the anti-gC1qR antibody failed to inhibit either of these parameters. Subsequently, we found that although this antibody inhibited C. albicans-induced phosphorylation of EGFR in primary human oral epithelial cells, it had no effect on primary mouse oral epithelial cells. The probable explanation for these results is that gC1qR is dispensable for the fungus to activate EGFR in mouse epithelial cells. Although it is also possible that the anti-gC1qR antibody used in the mouse studies did not block the association of gC1qR with EGFR in mouse cells, this seems unlikely because siRNA knockdown of EGFR in mouse oral epithelial cells also failed to inhibit EGFR phosphorylation in response to C. albicans.

We also found that although siRNA knockdown of IFITM3 inhibited the endocytosis of C. albicans by both the human OKF6/TERT-2 oral epithelial cell line and primary human oral epithelial cells, it had no significant effect on primary mouse oral epithelial cells. Collectively, these data suggest that two components of the EGFR signaling pathway, gC1qR and IFITM3, function differently in human versus mouse oral epithelial cells.

gC1qR is exploited by multiple microbial pathogens. gC1qR mediates the adherence of Plasmodium falciparum and Staphylococcus aureus to vascular endothelial cells and the adherence of Bacillus cereus spores to epithelial cells (44, 45, 56, 57). It is also an epithelial cell receptor for the L. monocytogenes internalin B (InlB) invasin and mediates the endocytosis of the organism (28). Because gC1qR lacks a transmembrane sequence, it is thought to induce endocytosis by signaling via another cell surface receptor. In the case of L. monocytogenes, the second receptor appears to be Met, the hepatocyte growth factor receptor (58). In some cell lines, gC1qR and Met have been found to function cooperatively to mediate the endocytosis of this bacterium (59). Although the primary amino acid sequence of the C. albicans Als3 invasin shares no homology with InlB, we have demonstrated that Als3 also interacts with gC1qR. Instead of Met, EGFR is the second receptor that associates with gC1qR and transduces the epithelial cell response C. albicans. Not only is gC1qR required for EGFR-mediated endocytosis of the fungus, but it is also necessary for the maximal epithelial cell inflammatory response to this organism. We have identified additional host cell receptors for C. albicans, including E-cadherin, HER2, the platelet-derived growth factor BB, the aryl hydrocarbon receptor, and gp96 (12–14, 18, 60). Whether gC1qR also interacts with these receptors remains to be determined.

## MATERIALS AND METHODS

### Epithelial cells and fungal strains.

The OKF6/TERT-2 oral epithelial cell line was provided by J. Rheinwald (Dana-Farber/Harvard Cancer Center, Boston, MA) and cultured as outlined previously (14, 17). Primary oral mucosal epithelial cells from humans and BALB/c mice were obtained from Cell Biologics, Inc., and grown following the manufacturer’s instructions. NIH/3T3 cells that expressed human EGFR and HER2 were provided by Nadege Gaborit (Institut de Researche en Cancérologie de Montpellier, France) and grown as described previously (48). The C. albicans wild-type strain SC5314 and the *als1*Δ/Δ, *als3*Δ/Δ, and *ece*1Δ/Δ mutants (16) were grown in yeast extract-peptone-dextrose (YPD) broth in a shaking incubator at 30°C for 18 h, after which the cells were pelleted by centrifugation and washed twice with phosphate-buffered saline (PBS). Yeast cells were suspended in PBS, diluted, and counted with a hemacytometer.

### Immunoprecipitation.

OKF6/TERT-2 oral epithelial cells were grown to confluence in 75-cm^2^ tissue culture flasks, and the culture medium was changed to KSF medium without supplements (Thermo Fisher Scientific; number 17005042) the night before the experiment. The next morning, cells were incubated for 90 min with supplement-free KSF medium containing 10^8^ C. albicans yeast cells or for 5 min with 50 ng/ml EGF. Cells incubated with fresh supplement-free KSF alone were processed in parallel as a negative control. After incubation, the medium was aspirated, and the cells were washed once with ice-cold PBS with Ca^2+^ and Mg^2+^ (PBS^++^). The proteins were cross-linked by incubation with 4% paraformaldehyde for 10 min. After rinsing the cells twice with ice cold PBS^++^, they were detached from the culture flasks with a cell scraper and collected by centrifugation. The cell pellet was lysed with 5.8% octyl-β-d-glucopyranoside (VWR; number 97061-760) with sonication. The lysate was clarified by centrifugation and then precleared with protein A/G magnetic beads (Thermo Fisher Scientific; number PI88802) for 30 min at 4°C. The precleared cell lysates were incubated with an anti-EGFR antibody (Santa Cruz Biotechnology; number SC-101, clone R-1) for 1 h at 4°C and precipitated with protein A/G magnetic beads for 2 h at 4°C. After the beads were washed 3 times with 1.5% octyl-β-d-glucopyranoside, the proteins were eluted with 8 M urea and stored at −80°C.

### Mass spectrometry.

The protein samples were treated with Tris (2-carboxyethyl) phosphine hydrochloride (Thermo Fisher Scientific; number 20491) and 2-chloroacetamide (catalog number 154955) for reduction and alkylation, respectively. Tris buffer (0.1 M [pH 8.5]) was added to each sample to decrease the urea concentration to a final concentration of 2 M. After dilution, the samples were digested with lysyl endopeptidase (Fujifilm Wako Chemicals; number 125-05061) and trypsin (Thermo Fisher Scientific; number 90058) for 20 h at 37°C. After quenching the reaction by adjusting the pH to 3, the digested samples were desalted using C_18_ columns (Thermo Fisher Scientific; number 89870) and then dried by vacuum centrifuge.

For the LC-MS/MS analysis, the samples were dissolved in 0.2% formic acid (FA) solution. The analysis was performed using an EASY-nLC 1000 (Thermo Fisher Scientific) connected to a Q Exactive Orbitrap mass spectrometers (Thermo Fisher Scientific). Solvent A consisted of 99.9% H_2_O and 0.1% FA, and solvent B consisted of 19.9% H_2_O, 80% acetonitrile, and 0.1% FA. Each sample was loaded onto an Easy Spray column (25 cm by 75 μm, 2-μm C_18_, ES802; Thermo Fisher Scientific) and separated over 90 min at a flow rate of 0.5 μl/min with the following gradient: 2 to 35% B (75 min), 35 to 85% B (5 min), and 85% B (10 min). The full MS scan was acquired at 70,000 resolution with a scan range of 350 to 2,000 *m/z*, an automatic gain control target of 1 × 10^6^, and a maximum injection time of 100 ms. The dd-MS2 scan was acquired at 17,500 resolution with a scan range of 200 to 2,000 *m/z*, an automatic gain control target of 5 × 10^4^, a maximum injection time of 64 ms, and an isolation window of 2.0 *m/z*. The proteomic data processing was performed using Proteome Discoverer 1.4 (Thermo Fisher Scientific) and the Sequest HT search engine. The search allowed for a precursor mass tolerance of 10 ppm, a minimum peptide length of 6, and a minimum peptide sequence number of 1.

### Proximity ligation assay.

To determine if the selected proteins associated with EGFR in intact epithelial cells, OKF6/TERT-2 cells were grown to confluence on fibronectin-coated coverslips. The cells were infected with 3 × 10^5^ C. albicans yeast cells in supplement-free KSF medium. After 90 min, the medium was aspirated, and the cells were fixed with 4% paraformaldehyde for 10 min. The coverslips were washed 3 times with PBS, after which the epithelial cells were permeabilized with 0.1% Triton X-100 in PBS for 20 min. The association between EGFR and WBP2, GBP6, TOLLIP, EEA1, DSG3, IFITM3, or gC1qR was detected using the Duolink In Situ Red Starter kit Mouse/Rabbit (Sigma-Aldrich; number DUO92101-1kit) according to the manufacturer’s instruction. The antibodies used were rabbit anti-EGFR (Genetex; number GTX121919, clone N1-2), mouse anti-EGFR (Santa Cruz Biotechnology; number SC-101, clone R-1), anti-WBP2 (Santa Cruz Biotechnology; number SC-514247, clone D-12), anti-GBP6 (Sigma-Aldrich; number HPA027744), anti-TOLLIP (Proteintech; number 117315-1), anti-EEA1 (Santa Cruz Biotechnology; number SC-365652 clone E-8), anti-DSG3 (Santa Cruz Biotechnology; number SC-53487, clone 5g11), anti-IFITM3 (Proteintech; number 11714-1), and gC1qR (Santa Cruz Biotechnology; number SC-23885, clone 74.5.2). The cells were imaged by confocal microscopy, and z stacks of the images were constructed using the Leica Application Suite X software (Leica).

### siRNA.

To knock down the levels of selected proteins, the OKF6/TERT-2 cells were grown in 6-well tissue culture plates to 50 to 80% confluence overnight. The next morning, the cells were transfected with EGFR (Santa Cruz Biotechnology; number SC-29301), WBP2 (Santa Cruz Biotechnology; number SC93955), TOLLIP (Dharmacon; number L-016930-00-0005), IFITM3 (Santa Cruz Biotechnology; number SC-97053), gC1qR (Santa Cruz Biotechnology; number SC-42880), or control (Qiagen; number 1027281) siRNA using Lipofectamine RNAiMAX (Invitrogen; number 13778150) following the manufacturer’s instructions. After 24 h posttransfection, the cells were seeded onto fibronectin-coated coverslips and incubated for another 24 h before use in the experiments. The extent of protein knockdown was determined by immunoblotting, and total loading was detected by probing the blots with an anti-GAPDH (anti-glyceraldehyde-3-phosphate dehydrogenase) antibody (Cell Signaling; number 5174, clone D16H11).

### Epithelial cell endocytosis and cell-association.

Our standard differential fluorescence assay was used to measure the number of C. albicans cells that were endocytosed by and cell associated with the OKF6/TERT-2 epithelial cells, primary mouse and human oral epithelial cells, and NIH/3T3 cells (13, 18, 22). The host cells were infected with 10^5^ C. albicans yeast cells. To ensure similar levels of endocytosis among the different host cells, the incubation times were 2.5 h for OKF6/TERT-2 cells and primary human oral epithelial cells, 2 h for the mouse oral epithelial cells, and 1.5 h for the NIH/3T3 cells. In the siRNA experiments, the transfected epithelial cells were seeded onto fibronectin-coated glass coverslips 24 h before infection. For experiments using gefitinib (1 μM; Selleck Chem, Inc.; number S1025) or the anti-gC1qR antibodies (10 μg/ml), the inhibitor or antibodies were added to the host cells 1 h prior to infection, and they remained in the medium for the duration of the experiment. All experiments performed in triplicate at least three times.

### Lentivirus construction and production and host cell transduction.

The transfer vectors (pLenti-EF1A-EGFP-BLAST or pLenti-EF1A-hC1QBP[NM_001212.4]-BLAST) were constructed by cloning enhanced green fluorescent protein (eGFP) or hC1QBP[NM_001212.4] into pLenti-Cas9-BLAST (Addgene; number 52962) at the BamHI and XbaI sites. The virus was produced by transfecting HEK293T cells with plasmid psPAX2 (Addgene; number 12260), plasmid pCMV-VSVG (Addgene; number 8454), and transfer vector (pLenti-EF1A-EGFP-BLAST or pLenti-EF1A-hC1QBP[NM_001212.4]-BLAST) using the X-tremeGENE 9 DNA transfection reagent (Sigma-Aldrich; number 6365787001) according to the manufacturer’s instructions. The supernatant containing the virus was collected at 60 h posttransfection, passed through a 0.45-μm-pore polyvinylidene difluoride (PVDF) filter, and stored at 4°C (short term) or −80°C (long term).

For transduction, the NIH/3T3 mouse fibroblast cells (untransformed control cells or the human EGFR/hHER2-transformed cell line [48]) were seeded into a 6-well plate in Dulbecco’s modified Eagle’s medium (DMEM) plus 10% bovine calf serum. The cells were transduced with lentivirus in the presence of 0.5 μg/ml Polybrene (Santa Cruz Biotechnology; number SC134220). The plates were centrifuged at 1,000 × *g* for 30 min and then incubated at 37°C in 5% CO_2_ overnight. The next morning, the cells were transferred to 10-cm-diameter tissue culture dishes. Two days postransduction, 10 μg/ml of blasticidin (Gibco; number A1113903) was added to the medium to select for transduced cells, and selection was maintained for 7 days. The successful transduction of eGFP was determined by fluorescence microscopy, and transduction of hC1QBP (gC1qR) was verified via immunoblotting with an anti-gC1qR antibody (clone 74.5.2) and an anti-EGFR antibody (Cell Signaling Technology; number 4267, clone 38B1).

### Receptor phosphorylation.

Analysis of the phosphorylation of EGFR and EphA2 was performed as previously described (11, 16). Briefly, the oral epithelial cells were seeded onto 24-well tissue culture plates and incubated overnight in supplement-free KSF medium. The next morning, the cells were treated with gefitinib, an anti-gC1qR antibody (clone 74.5.2), or control mouse IgG (R&D Systems; number MAB002) for 1 h. The cells were then stimulated with 10^6^ C. albicans yeast cells for 90 min, 40 μM candidalysin (Biomatik) for 5 min, or 1 ng/ml EGF for 5 min in the presence of the inhibitor or antibody. Next, the cells were lysed with 100 μl 2× SDS loading buffer in the present of phosphatase inhibitor cocktail (Sigma-Aldrich), protease inhibitor cocktail (Sigma-Aldrich), and phenylmethylsulfonyl fluoride (PMSF) (Sigma-Aldrich). After the samples were denatured at 90°C for 2 min, they were clarified by centrifugation. The proteins were separated by SDS-PAGE and transferred to PVDF membranes. Phosphorylated EGFR (Tyr 1068) was detected with a phospho-specific antibody (Cell Signaling Technology; number 2234) and enhanced chemiluminescence. Total EGFR was detected with an anti-EGFR antibody (Cell Signaling Technology; number 4267). Phosphorylation of EphA2 (Ser 897) was detected with a phospho-specific anti-EphA2 antibody (Cell Signaling Technology; number 6347, clone D9A1), and total EphA2 was detected with an anti-EphA2 antibody (Cell Signaling Technology; number 6997, clone D4A2). The phosphorylation of EGFR in mouse oral epithelial cells was determined similarly, except that total EGFR was detected with anti-EGFR antibody from Santa Cruz Biotechnology (number SC373746, clone A-10). All experiments were repeated at least three times.

### C. albicans association with epithelial cell gC1qR and EGFR.

The capacity of anti-gC1qR and anti-EGFR antibodies to block the association of C. albicans with gC1qR and EGFR was determined using our previously described method (61). Confluent OKF6/TERT-2 epithelial cells in a 6-well tissue culture plate were switched to supplement-free KSF medium the night before the experiment. The next morning, the epithelial cells were incubated with 10 μg/ml of control mouse IgG, an anti-gC1qR antibody (clone 74.5.2), or an anti-EGFR antibody (cetuximab; Lilly) for 1 h, and then infected with 6 × 10^6^ C. albicans blastospores. After 90 min of infection, the cells were detached with a cell scraper and collected by centrifugation. The epithelial cells were lysed by incubation with 5.8% *n*-octyl-β-d-glucopyranoside in PBS^++^ in the present of protease inhibitors and PMSF on ice for 1 h. The samples were centrifuged at 5,000 × *g* for 1 min, and the supernatants containing the total cell lysates were collected. The pellets, which contained the intact C. albicans cells and the epithelial cell proteins that were associated with them, were washed three times with cold 1.5% *n*-octyl-β-d-glucopyranoside in PBS^++^ containing protease inhibitors. The proteins that remained associated with the C. albicans cells were eluted by incubation with 6 M urea for 30 min on ice. After pelleting the organisms by centrifugation, the eluted proteins in the supernatants were separated by SDS-PAGE. The presence of gC1qR and EGFR in the eluates was determined by immunoblotting with an anti-gC1qR antibody (Abcam; number ab24733, clone 60.11) and an anti-EGFR antibody (clone 38B1). The experiment was repeated four times.

### Cytokine and chemokine measurement.

To measure the production of IL-1β and IL-8 by oral epithelial cells, the OKF6/TERT-2 cells were grown in 48-well tissue culture plates overnight in supplement-free KSF and then infected with 6.25 × 10^5^ C. albicans yeast cells for 8 h. The conditioned medium was collected and clarified by centrifugation. The levels of IL-1β and IL-8 in the conditioned medium were measured by ELISA using the human IL-1β DuoSet (R&D Systems; number DY20105) and the human IL-8 set (BD Biosciences; number BD555244).

The production of IL-1β, IL-8, IL-1α, and GM-CSF by the epithelial cells was measured similarly, except that the epithelial cells were grown in 24-well tissue culture plates and infected with 1.5 × 10^6^ C. albicans yeast cells. The levels of the inflammatory mediators were measured by Luminex Multiplex (R&D Systems; number LXSAHM-06). The experiments were repeated three times in duplicate.

### Mouse experiments.

Our standard immunocompetent mouse model of oropharyngeal candidiasis was used to assess the effects of an anti-gC1qR antibody and gefitinib on the infection (11, 16, 62). Immunocompetent BALB/c mice were administered 100 μg of the anti-gC1qR antibody (clone 74.5.2) by intraperitoneal injection on day −1 relative to infection. Control mice were injected with a similar amount of mouse IgG. Gefitinib was administered by adding the drug to powdered chow to a final concentration of 200 ppm, starting at day −2 relative to infection and continuing throughout the experiment. On the day of the infection, the mice were sedated, and a calcium alginate swab saturated with 2 × 10^7^ C. albicans yeast cells was placed sublingually for 75 min. After 1 day of infection, the mice were sacrificed, and the tongues were excised, weighed, and homogenized. The oral fungal burden was determined by quantitative culture of an aliquot of the homogenates, and the level of MPO in the homogenates was determined by commercial ELISA (Hycult Biotech; number HK210-02). The experiment was repeated twice using 4 to 5 mice per condition, and the results were combined.

### Statistical analysis.

The *in vitro* data were analyzed using one-way analysis of variance with Dunnett’s test for multiple comparisons. The mouse data were analyzed by the Kruskal-Wallis test with Dunn’s test for multiple comparisons. *P* values of ≤ 0.05 were considered to be significant.
