# Supplementary material for: The Globular C1q Receptor Is Required for Epidermal Growth Factor Receptor Signaling during Candida albicans Infection
Source: mBio. 2021 Nov 2;12(6):e02716-21. doi: 10.1128/mBio.02716-21 (PMC8561387; doi:10.1128/mBio.02716-21)
Supplement: FIG S1 [file mbio.02716-21-sf001.pdf]

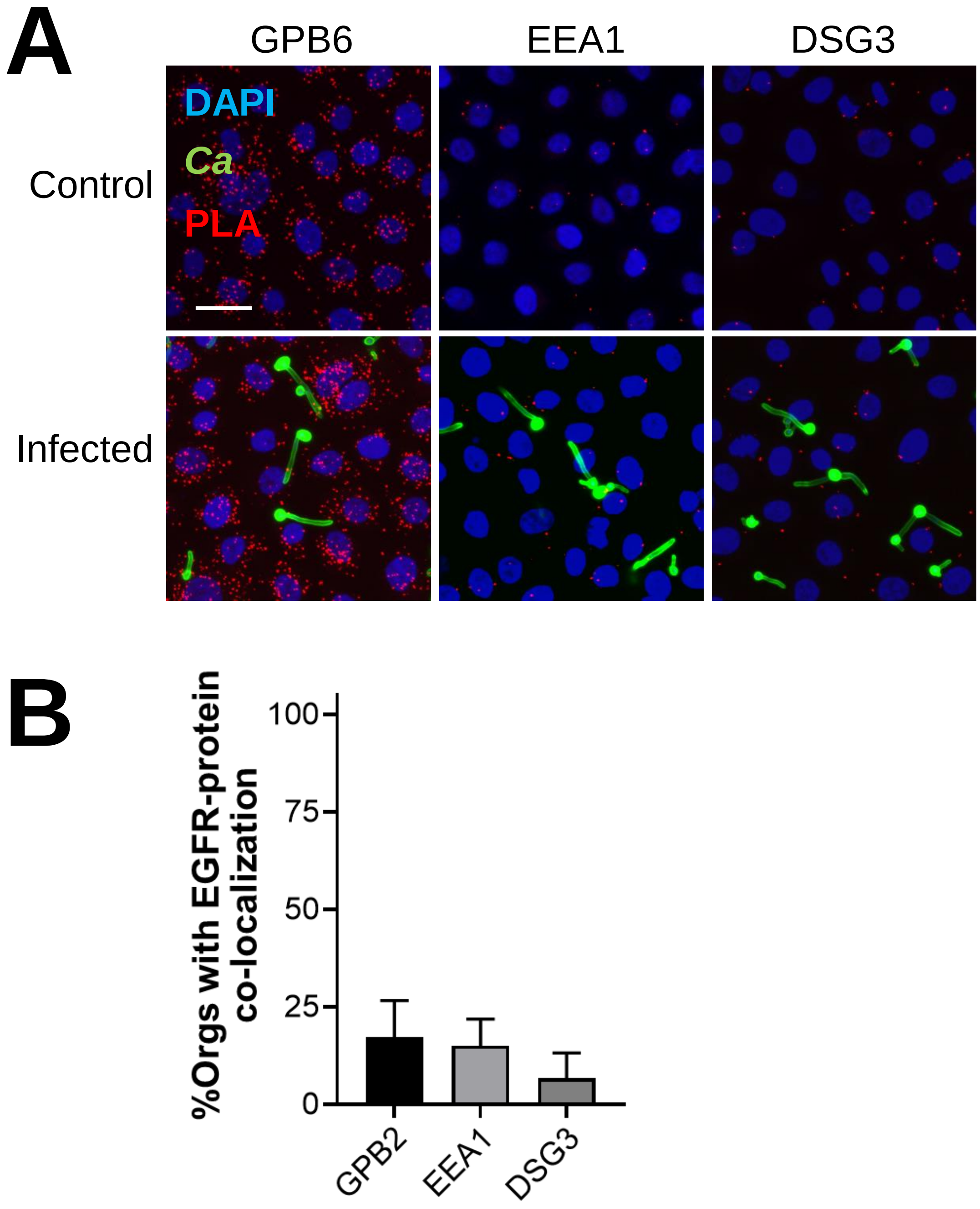

Fig. S1 Proximity ligation assays (PLA) to assess the physical association of the epidermal growth factor receptor (EGFR) with guanylate binding protein 6 (GBP6), early endosome antigen 1 (EEA1), and desmoglein-3 (DSG3) in the OKF6/TERT-2 oral epithelial cell line. (A) Confocal microscopic images of epithelial cells incubated in either medium alone (top) or infected with *C. albicans* (bottom) for 90 min. Red spots indicate the regions where the indicated proteins associate with EGFR. Results are representative of three independent experiments. Scale bar 25 µm. (B) Quantitative analysis of the images to determine the percentage of *C. albicans* cells with spots indicating the co-localization of EGFR with GPB2, EEA1, or DSG3. Results are mean ± SD of three independent experiments. Orgs, organisms.
