# Supplementary material for: The Globular C1q Receptor Is Required for Epidermal Growth Factor Receptor Signaling during Candida albicans Infection
Source: mBio. 2021 Nov 2;12(6):e02716-21. doi: 10.1128/mBio.02716-21 (PMC8561387; doi:10.1128/mBio.02716-21)
Supplement: FIG S2 [file mbio.02716-21-sf002.pdf]

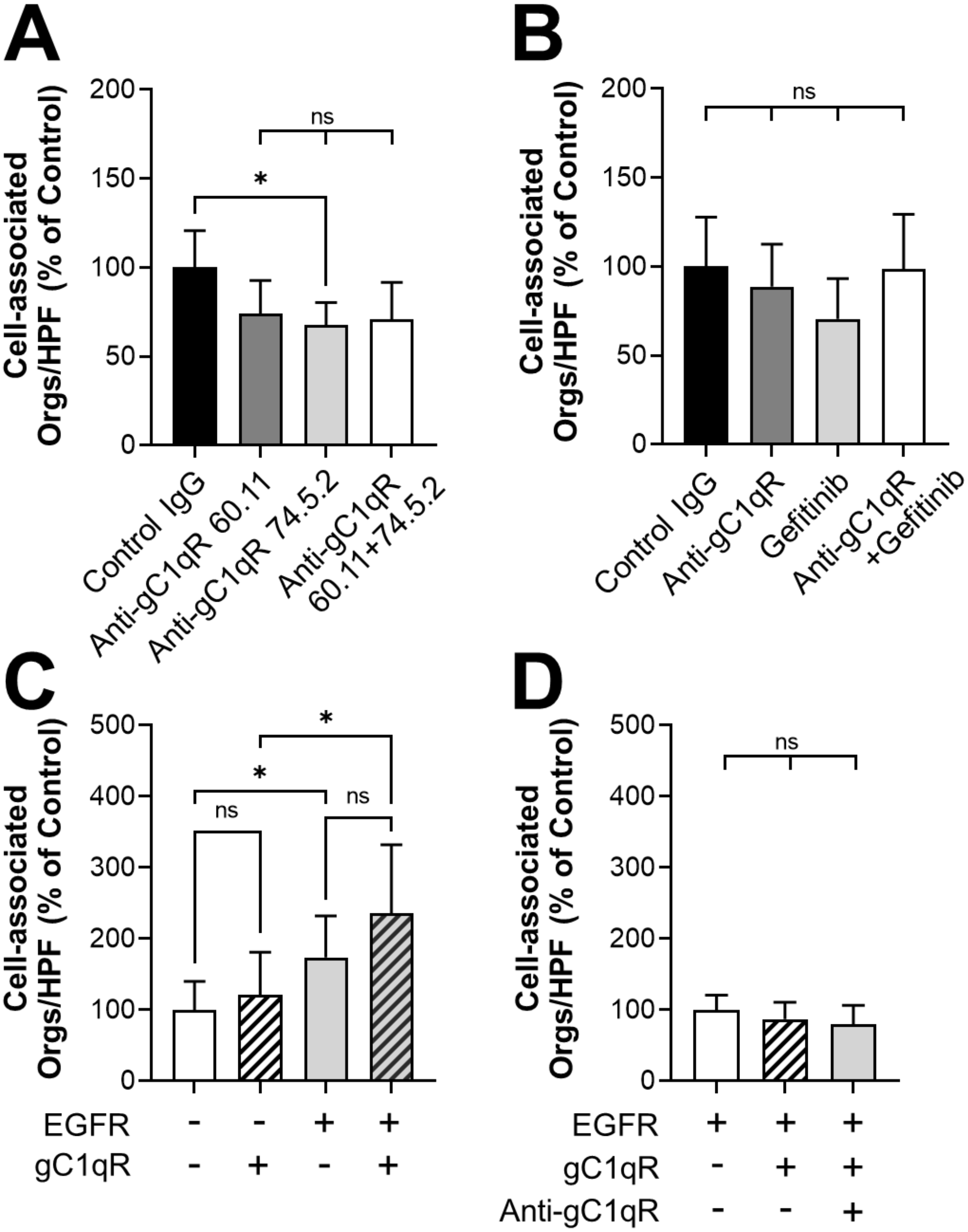

Fig. S2 Surface-expressed gC1qR has minimal effects on the number of *C. albicans* cells that are associated with oral epithelial cells. (A) Effects of two different anti-gC1qR monoclonal antibodies on the number of cell-associated *C. albicans* cells. (B) Effects of the anti-gC1qR antibody 74.5.2 and the EGFR kinase inhibitor, gefitinib on the number of cell-associated *C. albicans* cells. (C and D) Number of *C. albicans* cells that are cell-associated with NIH/3T3 cells expressing human gC1qR and/or human EGFR. (C) Effects of EGFR and gC1qR expression on cell-association. (D) Effects of inhibiting surface-expressed gC1qR with the anti-gC1qR antibody 74.5.2. Results are the mean ± SD of three independent experiments, each performed in triplicate. The data were analyzed using one-way analysis of variance with Dunnett's test for multiple comparisons. ns, not significant; Orgs/HPF, organisms per high power field;*$P < 0.05$.
