# Supplementary material for: The Globular C1q Receptor Is Required for Epidermal Growth Factor Receptor Signaling during Candida albicans Infection
Source: mBio. 2021 Nov 2;12(6):e02716-21. doi: 10.1128/mBio.02716-21 (PMC8561387; doi:10.1128/mBio.02716-21)
Supplement: FIG S3 [file mbio.02716-21-sf003.pdf]

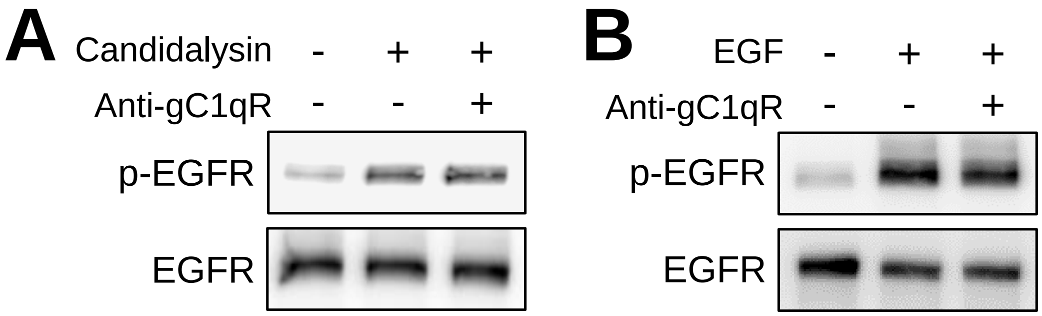

Fig. S3 Inhibition of gC1qR does not block EGFR phosphorylation in response to 40 µM candidalysin or 1 ng/ml epidermal growth factor (EGF). Representative Western blots from three independent experiments.
