# Supplementary material for: The Globular C1q Receptor Is Required for Epidermal Growth Factor Receptor Signaling during Candida albicans Infection
Source: mBio. 2021 Nov 2;12(6):e02716-21. doi: 10.1128/mBio.02716-21 (PMC8561387; doi:10.1128/mBio.02716-21)
Supplement: FIG S4 [file mbio.02716-21-sf004.pdf]

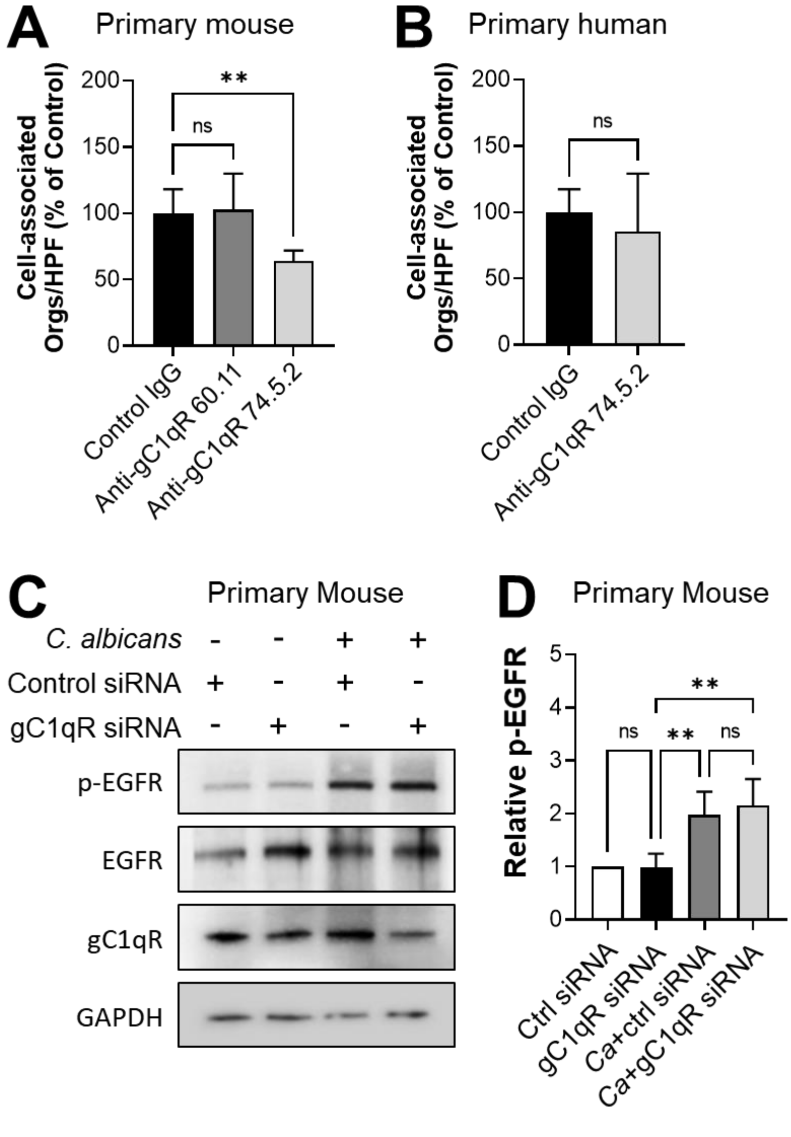

Fig. S4 Effects of anti-gC1qR antibodies and gC1qR siRNA on the interactions of *C. albicans* with human oral epithelial cells and primary mouse oral epithelial cells. (A and B) Effects of the indicated anti-gC1qR antibodies on adherence to primary mouse (A) and primary human (B) oral epithelial cells. (C and D) Effects of siRNA knockdown of gC1qR on the phosphorylation of EGFR in primary mouse oral epithelial cells. (C) Representative Western blot. (D) Densitometric analysis of four Western blots, such as the one shown in (C). Results in (A and B) are the mean ± SD of three independent experiments, each performed in triplicate. The data were analyzed using one-way analysis of variance with Dunnett's test for multiple comparisons. *Ca*, *C. albicans*; Ctrl, control; ns, not significant; Orgs/HPF, organisms per high power field; *$P < 0.05$, **$P < 0.01$.
