# Supplementary material for: The Globular C1q Receptor Is Required for Epidermal Growth Factor Receptor Signaling during Candida albicans Infection
Source: mBio. 2021 Nov 2;12(6):e02716-21. doi: 10.1128/mBio.02716-21 (PMC8561387; doi:10.1128/mBio.02716-21)
Supplement: FIG S5 [file mbio.02716-21-sf005.pdf]

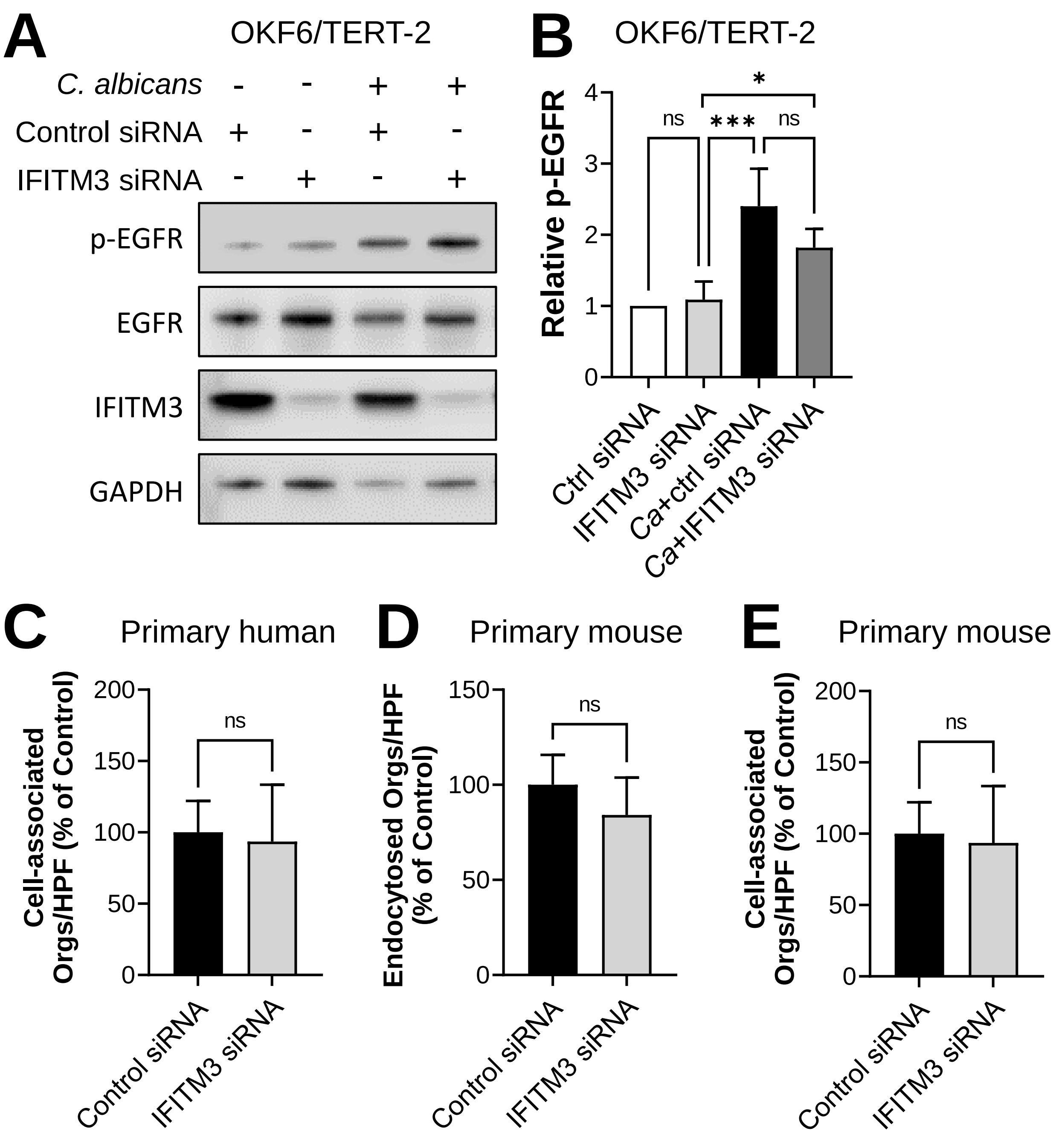

Fig. S5 Effects of siRNA knockdown of IFITM3 on the response of human and mouse oral epithelial cells to *C. albicans.* (A and B) Phosphorylation of EGFR in the human OKF5/TERT-2 oral epithelial cell line. (A) Representative Western blot. (B) Densitometric analysis of four Western blots, such as the one shown in (A). (C-E) Effects of siRNA knockdown of IFITM3 on the adherence of *C. albicans* to primary human oral epithelial cells (C), endocytosis by primary mouse oral epithelial cells (D), and adherence to primary mouse oral epithelial cells (E). Results in (C-E) are the mean ± SD of three independent experiments, each performed in triplicate. The data were analyzed using one-way analysis of variance with Dunnett's test for multiple comparisons. *Ca, C. albicans*; Ctrl, control; ns, not significant; Orgs/HPF, organisms per high power field; *$P < 0.05$, **$P < 0.01$.
